# *ExaFlexHH*: an exascale-ready, flexible multi-FPGA library for biologically plausible brain simulations

**DOI:** 10.3389/fninf.2024.1330875

**Published:** 2024-04-12

**Authors:** Rene Miedema, Christos Strydis

**Affiliations:** ^1^Department of Neuroscience, Erasmus Medical Center, Rotterdam, Netherlands; ^2^Quantum and Computer Engineering Department, Delft University of Technology, Delft, Netherlands

**Keywords:** brain simulation, FPGA, dataflow engine, systolic array, scalable, Inferior Olive, Hodgkin-Huxley, NeuroML

## Abstract

**Introduction:**

*In-silico* simulations are a powerful tool in modern neuroscience for enhancing our understanding of complex brain systems at various physiological levels. To model biologically realistic and detailed systems, an ideal simulation platform must possess: (1) high performance and performance scalability, (2) flexibility, and (3) ease of use for non-technical users. However, most existing platforms and libraries do not meet all three criteria, particularly for complex models such as the Hodgkin-Huxley (HH) model or for complex neuron-connectivity modeling such as gap junctions.

**Methods:**

This work introduces *ExaFlexHH*, an exascale-ready, flexible library for simulating HH models on multi-FPGA platforms. Utilizing FPGA-based Data-Flow Engines (DFEs) and the dataflow programming paradigm, ExaFlexHH addresses all three requirements. The library is also parameterizable and compliant with NeuroML, a prominent brain-description language in computational neuroscience. We demonstrate the performance scalability of the platform by implementing a highly demanding extended-Hodgkin-Huxley (eHH) model of the Inferior Olive using ExaFlexHH.

**Results:**

Model simulation results show linear scalability for unconnected networks and near-linear scalability for networks with complex synaptic plasticity, with a 1.99 × performance increase using two FPGAs compared to a single FPGA simulation, and 7.96 × when using eight FPGAs in a scalable ring topology. Notably, our results also reveal consistent performance efficiency in GFLOPS per watt, further facilitating exascale-ready computing speeds and pushing the boundaries of future brain-simulation platforms.

**Discussion:**

The ExaFlexHH library shows superior resource efficiency, quantified in FLOPS per hardware resources, benchmarked against other competitive FPGA-based brain simulation implementations.

## 1 Introduction

The observable dynamics of individual neurons are currently well-understood at a biophysical level. However, there is still much to be gained from studying the behavior of large-scale brain networks. Specifically, it is not yet fully understood how the complex dynamics of these networks give rise to higher-order brain functions. Accordingly, simulations of these brain networks can provide new insights into brain workings and human behavior (Murray et al., 2018; Lam et al., 2022). Furthermore, it is believed that brain-network research can also lead to better understanding of treatments for psychiatric disorders (Murray et al., 2018; Einevoll et al., 2019). For a comprehensive understanding of the brain, information from multiple scales is required. Simulations of detailed neural models of (large regions of) the human brain, which comprise around 86 billion neurons and 1 trillion synapses, based on latest estimates, are nowadays recognized to call for *exascale computing* (Amunts and Lippert, 2021).

**Figure 1:**
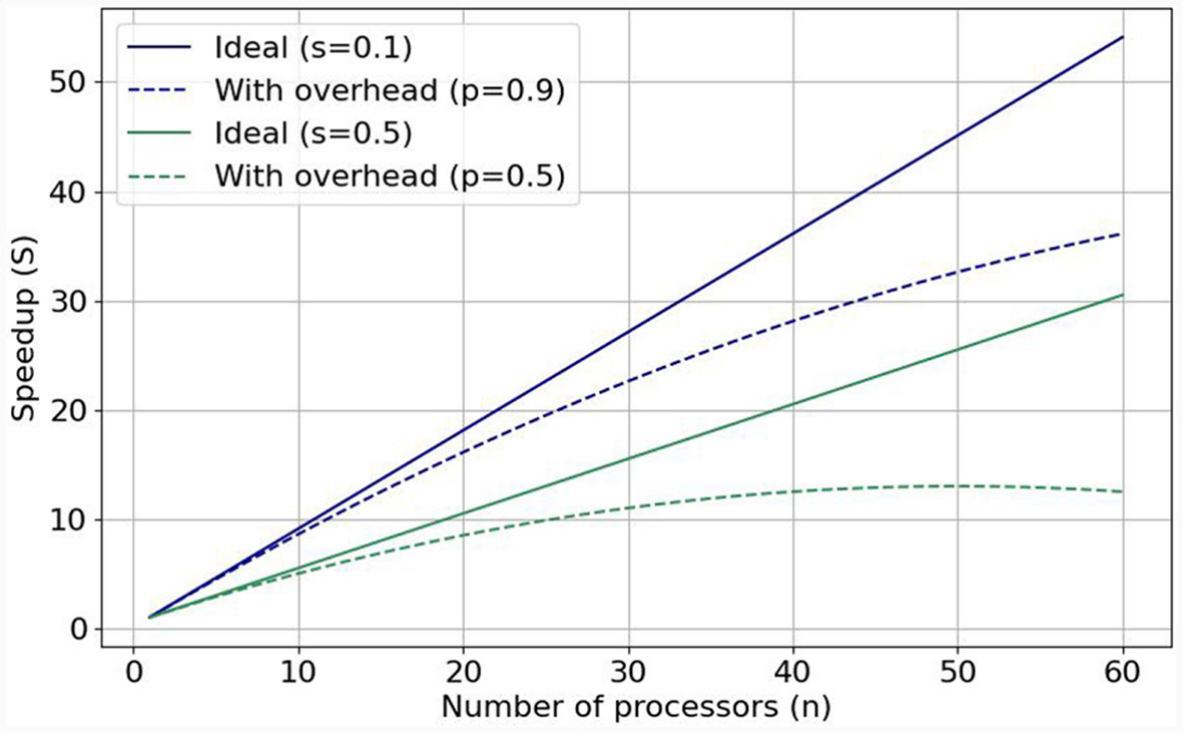
Visualization of Gustafson's law and weak scaling: with predicted (dotted lines, realistic) and without (solid lines, ideal) added communication overhead when the parallelizable fraction is equal to 0.9 and to 0.5.

Present-day High-Performance Computing (HPC) solutions have already delivered impressive brain simulations, however, their limitations become manifest once the *challenges* of simulating life-sized brain models are identified:

1. **Performance and scalability**. The computational power of a single processing unit plays a vital role in a simulation's overall performance. Therefore, HPC accelerators, which pack substantial computational throughput, are essential brain-simulation components. However, performance efficiency is equally crucial to pure performance, since we know that a single processing unit (or accelerator) cannot suffice for simulating the whole brain. The solution is to enlist more processing units to the simulation effort. The speedup gained from adding more processing units correlates with a program's inherent parallelism. Gustafson's Law (Gustafson, 1988) stipulates that significant speedup can be achieved with additional computational resources the higher the parallelizable portion (0 ≤ *p* ≤ 1) of the program is. Figure 1 shows speedups when enlisting increasing numbers of processing units for *p* = 0.9 (solid blue line) and *p* = 0.5 (solid green line). But these are ideal speedups in the absence of overheads that can bring system performance (efficiency) down. Nowadays, the so-called Memory-Wall problem (McKee, 2004) has emerged as a main challenge limiting achievable speeds; that is, memory speeds cannot keep up with accelerator speeds, effectively constraining achievable performance. This can be seen in the respective dotted lines in Figure 1, which represent sub-linear performance scaling. To tackle this issue, it is crucial to minimize memory accesses and keep data as close to the processing unit as possible. Over networks of processing units, the problem becomes even more pronounced, especially in the case of simulating large-scale and realistic brain models that exhibit dense synaptic activity among their nuclei. That is why—besides enlisting powerful accelerators—it is imperative to also implement low-latency and high-throughput interconnects to ensure good *performance scalability* and efficient utilization of all computational resources, minimizing memory accesses (Ishii et al., 2017). This is exemplified in cases such as Pronold et al. (2022), where network communication dominates simulation time, and communication time deteriorates with enlisting more CPU MPI processes.
2. **Flexibility**. Simulator flexibility is a crucial property of modern-day simulation platforms since the computational-neuroscience community is in constant flux, always tweaking model aspects and tuning their parameters. Consequently, brain simulators require sufficient flexibility and modularity in order to cover a wide variety of configurations needed for research.
3. **Usability**. For all their complexity, mounting simulations should be as easy to use as possible for neuroscientists. What is more, if an HPC simulator should make use of hardware accelerators to improve its performance, as is the current trend, then harnessing its full potential should–ideally–not require specialized knowledge from an acceleration expert working next to the neuromodeler.

In this work, we advocate the use of exascale-ready computing methods for facilitating the steep requirements of large-scale brain simulations. Traditional HPC solutions are known to fall short of meeting these requirements. To eschew the inherent memory bottleneck of conventional (von-Neumann) processing technologies such as Central Processing Units (CPUs) and Graphics Processing Units (GPUs), Field-Programmable Gate Array (FPGA) acceleration is recognized as one of the most robust platforms for attaining scalable performance when discounting exotic approaches such as quantum computing. Until now, their low usability (which is neurosimulator *challenge 3*) has been the main deterrent neuromodelers consistently adopting them in the field. In this work, we will demonstrate that this final barrier can be largely overcome through the combination of key enabling technologies and special design methods.

In terms of *methods*, firstly, the use of modern FPGAs allows designing dataflow-computing kernels instead of following the typical control-flow (i.e., von-Neumann) approach, which results in significant performance gains for data-intensive workloads, such as brain simulation (Flynn et al., 2013). Building on top of this dataflow-computing paradigm, parameterizable FPGA libraries such as *flexHH* (Miedema, 2019) have been proposed for simulating a gamut of biologically plausible brain models. The trivial control flow of dataflow kernels, in turn, permits the design of very simplified hardware interconnects across FPGA accelerators, effectively leading to communication-efficient, systolic-array-like multi-FPGA ensembles. Thus, on the *technology* front, some dataflow-enabled FPGA platforms can offer the option for direct communication links between them, allowing for direct, low-latency, and high-throughput connections, without the interference of a host CPU. This makes them highly promising for exhibiting good performance scalability (Pell et al., 2013). Finally, the addition of latest High-Bandwidth Memory (HBM) modules on FPGA chips significantly improves their performance for memory-bound applications (Wang et al., 2020).

The aforementioned aspects point to specific multi-FPGA platforms as a highly promising candidate for tackling the three identified challenges for biologically detailed, exascale-ready, large-scale brain simulations. In this article, we present *ExaFlexHH*, a dataflow-based, performance-scalable, and user-friendly brain simulation library. Our contributions are as follows:

- A concrete proposal for attaining exascale-ready brain simulations based on an ensemble of cutting-edge technologies and methods.
- A review and taxonomy of multi-accelerator platforms for brain simulations.
- A future-proof, scalable, multi-FPGA simulation library called *ExaFlexHH*, for eHH models that is synthesis-free, parameterizable, and flexible.
- A detailed performance evaluation of *ExaFlexHH*, including a performance model for making future, scale-out projections.

The remainder of this paper is organized as follows: Related works are presented in Section 2. In Section 3, we provide crucial background information and detail the ExaFlexHH implementation. In Section 4, we present our evaluation results. Section 5 examines performance bottlenecks and evaluates the potential for improvement if some of these bottlenecks are addressed. Finally, in Section 6, we present our conclusions.

## 2 Related work

Many HPC works have aimed at brain research in recent years. We have chosen only works that meet the following criteria: firstly, works that utilize high-performance accelerators since these are a crucial component in achieving the large-scale and highly scalable simulations required for brain research. Secondly, works that utilize multi-accelerator computation, since this is essential for achieving the required performance density for large-scale and highly detailed brain simulations. We have, thus, excluded SpiNNaker and NEST (Gewaltig and Diesmann, 2007) due to the absence of accelerator support as well as Brain2 (Stimberg et al., 2019) and GeNN (Yavuz et al., 2016) due to a lack of support for scale-out acceleration. Finally, platforms focused on machine learning and cognitive neuroscience are excluded as generally unsuitable for simulating biologically detailed models; therefore, BiCoSS (Yang et al., 2021), Loihi (Davies et al., 2018), and Tianjic (Deng et al., 2020) have been dropped.

An overview of eligible works is shown in Table 1. We have organized information into three main categories, largely matching the three challenges set in the previous section, as follows: (I) **Performance (scalability):** The number of accelerators per node, the number of nodes, and the type of connections used will be specified and presented. This information will give insight into the potential performance that can be achieved and the cost of utilizing the system. (II) **User experience:** Computational-neuroscience language support such as PyNN (Davison et al., 2009) and NeuroML (Cannon et al., 2014). Also, advanced and easy-to-use graphical user interfaces (GUIs) are contributing to user experience. We distinguish three levels of flexibility: no ( ), partial (+) and full (++) flexibility. (III) **Biological plausibility:** The neuron-model(s) and type(s) of synapses are specified here. Compared to other types of models, HH and particularly eHH models incur high computational costs (Izhikevich, 2004; Kozloski and Wagner, 2011), but more crucially, also high communication costs due to the detailed modeling of electrical and chemical synaptic activity. Of the two, chemical-synapse activations are relatively slow events and can be simulated in an event-based manner, reducing communication costs. In contrast, electrical synapses (i.e., gap junctions) require continuous interneuron communication, stressing multinode-accelerator data transfers, which can limit throughput and latency (Hahne et al., 2015; Jordan et al., 2018).

**Table 1:** Comparison of *ExaFlexHH* with other brain simulation developments.

**Platform**	**Performance and scalability**				**User experience**		**Biological plausibility**
	**Accelerator type**	**Accel./Node**	**# Nodes**	**Connection type**	**User-friendly interface**	**Flexibility**	
BrainScaleS	Analog + Digital ASIC	1	1	Extoll architecture using 1-GEth*	+	+	AdEx & LIF, multiple compartments + chemical/electrical synapses
TrueNorth	Digital ASIC	1 or 16	16 or 1	1-GEth packet-switched network/ native inter-chip interfaces			LIF + chemical synapses
Multi-GPU Neural Simulator	GPU	2	1	Not mentioned		+	Izhikevich + chemical synapses
BSim	GPU	4	1	NVLINK using NVHS	+	++	LIF + chemical synapses
Spice	GPU	8	1	NVLINK		+	LIF + chemical synapses
CarlSim 4	GPU	4	1	Not mentioned	+	++	LIF & Izhikevich, multiple compartments + chemical/electrical synapses
BlueHive	FPGA	4	1	PCIe & SATA (bare metal)			Izhikevich + chemical synapses
SNAVA	FPGA	2	1	FPGA high-speed serial links	+		LIF & Izhikevich + chemical synapses
NeuroFlow	FPGA	6	1	FPGA links	+		LIF, AIF, Izhikevich, HH¶ + chemical synapses
NEURON/CoreNEURON	CPU/GPU	2	2/4	NVLINK	+	++	Everything
Arbor	CPU/GPU	1	128	Asynch., MPI-based spike comm.	+	++	Everything
ExaFlexHH *(this work)*	FPGA	8	1	FPGA direct links	†	+	HH + electrical synapses

¶ No results shown for HH models. † Only NeuroML-compatible, the parser only supports cell descriptions.

BrainScaleS-2 (Pehle et al., 2022), is an analog hardware platform for emulating spiking neural networks. It supports Adaptive-Exponential Integrate-and-Fire (AdEx) or integrate-and-fire (I&F) neurons and allows for multi-compartmental features (Kaiser et al., 2022), and conductance-based synapses. The platform utilizes digital chips for control and plasticity management and the EXTOLL network protocol (Neuwirth et al., 2015) in combination with FPGAs for interconnectivity. BrainScaleS-2 can be interfaced with through PyNN (Müller et al., 2022) providing a user-friendly interface. However, the platform has not yet been tested for performance scalability and consequently this remains uncertain. TrueNorth (Akopyan et al., 2015) is a specialized chip for neural simulations that has been demonstrated in different configurations such as a single chip on a NS1e board, 16 NS1e boards connected through a 1 GbE packet-switch network, and the NS16e platform on a 4 × 4 board. However, its performance scalability has not been evaluated making good scalability uncertain. Additionally, TrueNorth chips only support I&F models, limiting their biological plausibility. The Corelet developer kit (Amir et al., 2013) requires learning its environment, instead of using standardized languages such as NeuroML or PyNN. Both aforementioned platforms are Application-Specific Integrated Circuits (ASICs). ASIC solutions offer excellent performance, scalability and energy efficiency but lack the level of flexibility needed in the constantly evolving field of computational neuroscience. Even trivial model changes can easily result in a new development cycle, significantly delaying the research process and stacking costs. Consequently, such solutions do not meet the general brain-simulation needs.

In Thibeault et al. (2011), Qu et al. (2020), and Bautembach et al. (2021), three GPU-based simulation platforms for Spiking Neural Networks (SNNs) are introduced: a multi-GPU neural (mGPUns) simulator, BSim, and Spice. The scalability results of mGPUns are constrained as only the results of 1 and 2 GPUs are shown and thus insight is limited. Both the performance results of BSim and Spice have shown limited performance scalability, ranging from as low as 1.6 comparing 4-GPU execution to single-GPU with BSIM execution to ≈5.1 when using 8 GPUs compared to 1 GPU with Spice. Additionally, the simulators lack the support of HH-type models and only BSim supports PyNN. In contrast, CARLsim 4 (Chou et al., 2018) is a multi-GPU simulator that aims at supporting a wide range of neural models and synapse types including Izhikevich models, multiple compartments, and current and conductance-based synapses with plasticity. CARLsim 4 offers tools for parameter tuning and visualization and uses a custom API rather than standard languages like NeuroML or PyNN. However, performance evaluations show limited performance scalability as the maximum increase in performance is 1.95 × and 2.44 × when using 2 and 4 GPUs, respectively, compared to 1 GPU; also, there is no support for eHH models. Consequently, it is not optimally qualified for large-scale, biologically detailed simulations. Overall, all discussed GPU platforms exhibit excellent performance, flexibility, and usability. However, they are von-Neumann architectures with all that this entails, including thread synchronization, instruction overheads and memory latencies (Hameed et al., 2010; Yazdanpanah et al., 2013). These limitations become especially evident when energy efficiency is a consideration (Lant et al., 2019). While there have been promising developments such as NVLink, performance scalability is questionable due to difficulties in efficiently exploiting such developments (Li et al., 2019). Therefore, the GPU solutions are not considered optimal to achieve ideal performance scalability.

In the field of FPGAs, Bluehive (Moore et al., 2012) is a computing platform that utilizes 16 FPGAs in a rack, connected via PCIe and a custom PCIe-to-SATA adapter for a reconfigurable topology. It can simulate 64k Izhikivich neurons with 64 million synapses per FPGA in real-time on a four FPGA setup. However, the system does not provide any scalability results. Additionally, Bluehive is limited to Izhikevich models with no easy to add new functionalities and has a lack of a user-friendly interface reducing the accessibility for non-experts. SNAVA (Sripad et al., 2018) is an FPGA-based neural simulation platform with a custom interface for model selection and connectivity. While it can simulate leaky integrate-and-fire (LIF) and Izhikevich neurons, it lacks support for HH-type models and widely used languages like PyNN and NeuroML. Using fixed-point simulation for performance may impact accuracy in stiffer, biophysical models like eHH. Although it is designed for scalability with an expandable ring structure, experimental results are limited to a two-FPGA network, and model updates' impact on synthesis cycles introduces uncertainty and challenges SNAVA's flexibility. NeuroFlow (Cheung et al., 2016) is another multi-FPGA neural simulation platform with PyNN compatibility. It supports a range of models including HH neurons. This makes NeuroFlow one of the most user-friendly and complete neural simulation platforms available. However, it does not support gap-junction connectivity and multi-compartment models. Additionally, performance and scalability results are limited to the simpler Izhikevich models and event-driven implementations, with synapses between neurons on the same or neighboring FPGAs. Therefore, while NeuroFlow is promising, its performance and scalability for complex cases remain to be seen. In all FPGA solutions, the hardware is configured specifically for each application, delivering high performance, while also providing higher levels of (re)modeling flexibility as well as energy efficiency (Chow et al., 2012; Guo et al., 2012; Arram et al., 2013; Gan et al., 2013). Unfortunately, flexibility comes at the cost of notoriously low levels of programming ease compared to GPUs due to the stringent hardware-programming languages involved (e.g., VHDL, Verilog) as well as the large, hardware-synthesis debug cycles.

Finally, we include two full-fledged neurosimulator environments, which support multinode simulations using CPU-only or a mix of CPU & GPU implementations. The community standard simulator NEURON (Hines and Carnevale, 1997) integrates HPC solutions through CoreNEURON (Kumbhar et al., 2019; Awile et al., 2022). Therefore, it supports simulations on multi-threaded CPUs and GPUs, and multi-node processing through the use of MPI. This brings significant performance benefits to NEURON. However, its performance scalability is still far from ideal as it is constrained by the previously mentioned von-Neumann limitations. Arbor (Abi Akar et al., 2019) is a neural simulator focusing on high-performance processing and multi-compartmental neuron models, including eHH with gap junctions. Additionally, Arbor is designed to be user-friendly, providing an object-oriented interface. However, its performance scalability when modeling complex connectivity is unclear, and centralized spike exchange between neurons may limit its ability to scale efficiently. Furthermore, the capability to handle large-scale simulations with gap-junction connectivity is not demonstrated.

*ExaFlexHH* is a high-performance, hardware library for simulating biologically plausible eHH models on one or multiple FPGAs. The use of the dataflow paradigm allows for efficient utilization of hardware acceleration and support for multiple FPGAs connected within a single node in a ring structure allows for low-latency interconnects. The system's modular design allows easy modification of parameters without re-synthesis, while NeuroML compliance ensures user-friendliness. Though communication can extend seamlessly outside a single compute node, *ExaFlexHH* has been currently demonstrated on as many as 8 FPGAs on a single compute node, leaving multi-node as future work. Despite this limitation, *ExaFlexHH* provides a flexible, highly scalable, and high-performance option for the simulation of large-scale eHH models.

## 3 Method

This section begins with the discussion of HH-type models and our use case, the Inferior Olive, and an explanation of why this model is suitable as a benchmark in Section 3.1. The subsequent Section 3.2 discusses the Maxeler system and the dataflow paradigm. Then, in Section 3.3 the predecessor of *ExaFlexHH, flexHH* is discussed. Finally, the implementation is detailed in Section 3.4.

### 3.1 Hodgkin-Huxley-type models

The HH neural networks described here are represented by a set of Ordinary Differential Equations (ODEs). Therefore, an ODE solver is required to solve (i.e., simulate) these models. The simplest ODE solver is the forward-Euler, shown in Equation (1). Here, *u*^*n*^ represents the approximated state variables step *n*, Δ*t* denotes the time-step size, and *f* a describes the vector of state derivatives. This process progresses iteratively for a simulation.


(1) $$\begin{array}{l} u^{n+1}=u^{n}+\Delta t\cdot f\left(u^{n}\right) \end{array}$$

**Figure 2:**
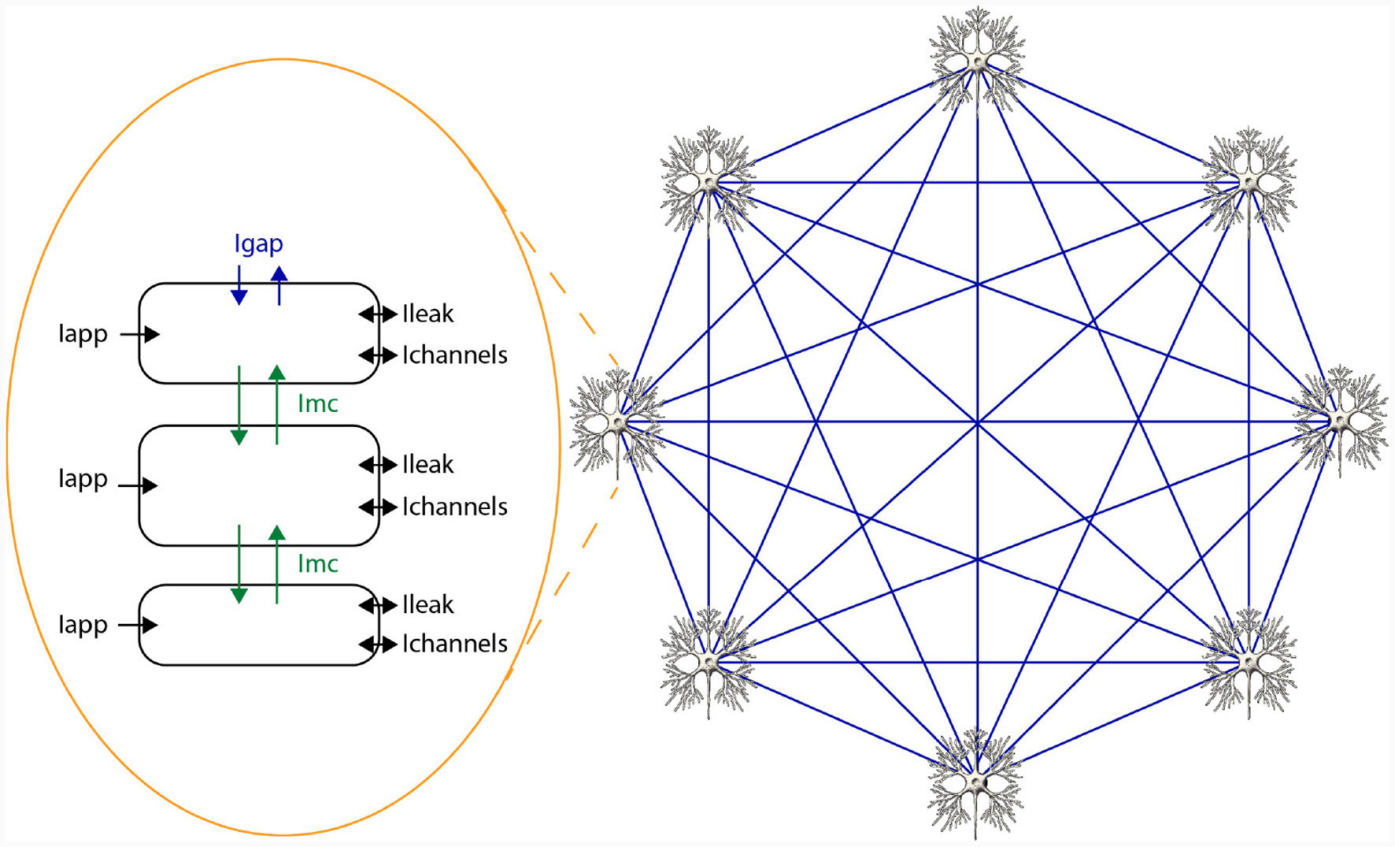
Schematic overview of a network of HH-type neurons.

In HH-type models, two types of state variables are involved: membrane voltages across cellular compartments, and gate variables indicating ion-channel openings. The voltage derivative for a specific compartment *i* in an HH-type model is computed as per Equation (2), where *C* signifies membrane capacitance, *I*_*app, i*_ is the applied current representing external stimuli, *I*_*channels, i*_ aggregates all ion-channel currents, *I*_*leak, i*_ indicates leakage current, and *I*_*mc, i*_ and *I*_*gap, i*_ reflect currents from inter-compartment connections and gap junctions, respectively. Notably, these latter terms are model-dependent and may be excluded if not applicable. For example, the original HH only consists of a single cell and a single compartment and therefore, does not include *I*_*mc, i*_ and *I*_*gap, i*_. A network of 3 compartmental neurons is given in Figure 2. This figure shows three compartments, the currents per compartment, and all-to-all, through gap junctions, connected network of 8 neurons.

*I*_*app, i*_ can be defined by any arbitrary function, while *I*_*channels, i*_ follows Equation (3). In this equation, *N*_*channels, i*_ is the number of channels for compartment *i*, *g*_*c, j*_ is the conductance, and *V*_*c, j*_ is the reverse voltage of channel *j*. Furthermore, *I*_*channels, i*_ involves *yProd*_*j*_, the product of gate activation variables of channel *j* calculated using Equation (4). In this equation *M*_*gates*[*j*]_ represents the amount of different gate types and *p*_*k*_ is an integer that counts the number of gates for a given type within the channel.


(2) $$\frac{dV_{i}}{dt}=\frac{I_{app,i}-I_{channels,i}-I_{leak,i}-\;I_{mc,i}-\;I_{gap,i}}{C}$$

(3) $$\begin{array}{l} I_{channels,i}=\sum_{j=0}^{N_{channels,i}-1}I_{channel,j}\\ \;=\sum_{j=0}^{N_{channels}-1}g_{c,j}(V-V_{c,j})yProd_{j} \end{array}$$

(4) $$\begin{array}{l} yProd_{j}=\overset{M_{gates\left[j\right]}-1}{\underset{k=0}{\prod }}y_{k}^{p_{k}} \end{array}$$

For models supporting multiple compartments, *I*_*mc, i*_ is added to represent the current between adjacent compartments. To calculate the current flowing between compartments, we use a similar equation as in Schweighofer et al. (1999), shown in Equation (5). This equation incorporates the number of linked compartments *N*_*comps, i*_ to compartment *i*, the internal conductance *g*_*int*_, the surface ratio of adjoining compartments *p*_*i, j*_, and their respective membrane voltages (*V*_*i*_, *V*_*j*_).


(5) $$\begin{array}{l} I_{mc,i}=g_{int}\overset{N_{comps,i}-1}{\underset{j=0}{\sum }}\frac{V_{i}-V_{j}}{p_{i,j}} \end{array}$$

Gap junctions, the inter-cellular connections, are modeled following a generalized approach from Schweighofer et al. (2004) by calculating *I*_*gap, i*_ through Equation (6), where *V*_*i, j*_ is the voltage difference between cell *i* and *j*, *c*_0_, *c*_1_, and *c*_2_ are identical between connections, and *w*_*i, j*_ represents the weight between compartments *i* and *j*, where *j* belongs to a different cell than *i*, therefore enhancing data efficiency and model adaptability.


(6) $$\begin{array}{ll} I_{gap,i} & =\overset{N_{cells}-1}{\underset{j=0}{\sum }}\left(w_{i,j}\left(c_{0}\exp\left(c_{1}\cdot V_{i,j}^{2}\right)+c_{2}\right)V_{i,j}\right) \end{array}$$

The derivatives of the gate-activation variables represented by *y*_*j*_ are also required and can be determined via Equation (7) and/or Equation (8). These involve transition rates α_*j*_ and β_*j*_ or the target value $\underset{j}{\inf}$ and the time constant τ_*j*_, which are generally determined by exponential functions. For reference, the derivatives of the gate-activation variables of the original HH model (Hodgkin and Huxley, 1952) are presented in Equations (9–17).


(7) $$\begin{array}{l} \frac{dy_{j}}{dt}=\left(1-y_{j}\right)\cdot \alpha_{j}-y_{j}\cdot \beta_{j} \end{array}$$

(8) $$\begin{array}{l} \frac{dy_{j}}{dt}=\frac{inf_{j}-y_{j}}{\tau_{j}} \end{array}$$

(9) $$\begin{array}{l} \frac{dn}{dt}=\alpha_{n}\left(1-n\right)-\beta_{n}n \end{array}$$

(10) $$\begin{array}{l} \frac{dm}{dt}=\alpha_{m}\left(1-m\right)-\beta_{m}m \end{array}$$

(11) $$\begin{array}{l} \frac{dh}{dt}=\alpha_{h}\left(1-h\right)-\beta_{h}h \end{array}$$

(12) $$\begin{array}{l} \alpha_{n}=\frac{0.01\left(V+10\right)}{\exp\left(\frac{V+10}{10}\right)-1} \end{array}$$

(13) $$\begin{array}{l} \beta_{n}=0.125\exp\left(V/80\right) \end{array}$$

(14) $$\begin{array}{l} \alpha_{m}=\frac{0.1\left(V+25\right)}{\exp\left(\frac{V+25}{10}\right)-1} \end{array}$$

(15) $$\begin{array}{l} \beta_{m}=4\exp\left(\frac{V}{18}\right) \end{array}$$

(16) $$\begin{array}{l} \alpha_{h}=0.07\exp\left(\frac{V}{20}\right) \end{array}$$

(17) $$\begin{array}{l} \beta_{h}=\frac{1}{\exp\left(\frac{V+30}{10}\right)+1} \end{array}$$

The model used to benchmark *ExaFlexHH* is a model of the Inferior Olive (IO) which is a brain region implicated in learning and online motor control (Schweighofer et al., 2013). De Gruijl et al. (2012) developed a model of an IO network employing an eHH description. The extensions incorporated in this model include more sophisticated ion gates, multiple compartments, and gap junctions. Specifically, each IO cell in the model consists of three compartments: the dendrite, soma, and axon. The sophisticated ion gates and the multi-compartmental structure augment the complexity as well as the computational demands belonging to the intracellular dynamics. Moreover, the inclusion of gap junctions among the dendrites, which represents instant, continuous interneuron connections, further intensifies the complexity by requiring communication among cells, therefore disrupting parallelism and posing a challenge to straightforward performance scaling. Given the biological plausibility, complexity, and computational requirements of this model, it is a fitting scenario for evaluating the *ExaFlexHH* framework. The equations used for the IO model are provided as Supplementary material. For an exhaustive description of the model, readers are directed to De Gruijl et al. (2012).

### 3.2 Maxeler system and dataflow paradigm

Neuron dynamical equations typically require minimal control, such as a few if/else statements, making them well-suited for the dataflow paradigm. This paradigm, especially when implemented using FPGAs, can be efficiently leveraged. Maxeler Technologies offers a unique solution in this space with its Data-Flow Engines (DFEs) and associated tools (Pell et al., 2013). The DFEs are FPGA-based hardware that are programmed via the use of Maxeler tools and excel in exploiting the dataflow paradigm.

In the dataflow paradigm, traditional control logic is mostly absent. Compute dependencies are resolved statically, at compile time. Control is effectively reduced to counters that advance data through execution units in the datapath. This approach allows for the majority of FPGA resources to be dedicated to computation rather than control logic. Moreover, it enables implementation in a deeply pipelined manner, significantly enhancing computational throughput.

A key factor for efficient dataflow implementation on FPGA-based hardware is the use of on-chip memory. Contemporary FPGAs, such as the Xilinx Ultrascale+ (AMD, 2023), feature three levels of on-chip memory. The first level, utilizing logic slices and lookup tables, creates flexible RAM but is not efficient for larger memories. The second level, Block Random-Access Memory (BRAM), comprises physical memory units with up to 36 Kbits storage capacity. These units can be combined for greater capacity. The third level, UltraRAM (URAM), offers the largest storage (288 Kbits) but is the least flexible. The Maxeler tools abstract these memories and collectively call them FMem (Fast Memory). With the use of the Maxeler tools, the on-chip memory is classified as FMem. Additionally, the DFEs contain on-board DRAM, ranging in the order of tens of gigabytes, which is referred to as LMem (Large Memory) in the Maxeler tools.

Another advantage of using the dataflow paradigm with DFEs is scalability. DFEs connect directly via the MaxRing, which is a low-overhead, wired Daisy-chain connection among DFEs in a server node and accommodated via using unused PCIe pins on the mainboard. The MaxRing, thus, offers high-bandwidth, low-latency, and highly scalable interconnects. This facilitates deeper pipeline designs, increased parallelism, and a highly scalable platform architecture that *ExaFlexHH* intends to harness.

Programming the Maxeler system involves three fundamental parts:

1. CPU-Host Code: Written in C, this code initializes data, coordinates DFEs, and manages I/O (Input/Output) between the CPU-host and DFEs.
2. Kernel Code: Using MaxJ, an extended version of Java, this code defines the functionality of the kernel(s) on the DFEs.
3. Manager: Also defined in MaxJ, the manager configures I/O for the kernels, including on-board DRAM, inter-kernel communication, MaxRing, and CPU-host interactions. It also sets hardware-specific configurations like frequency and synthesis strategies.

**Figure 3:**
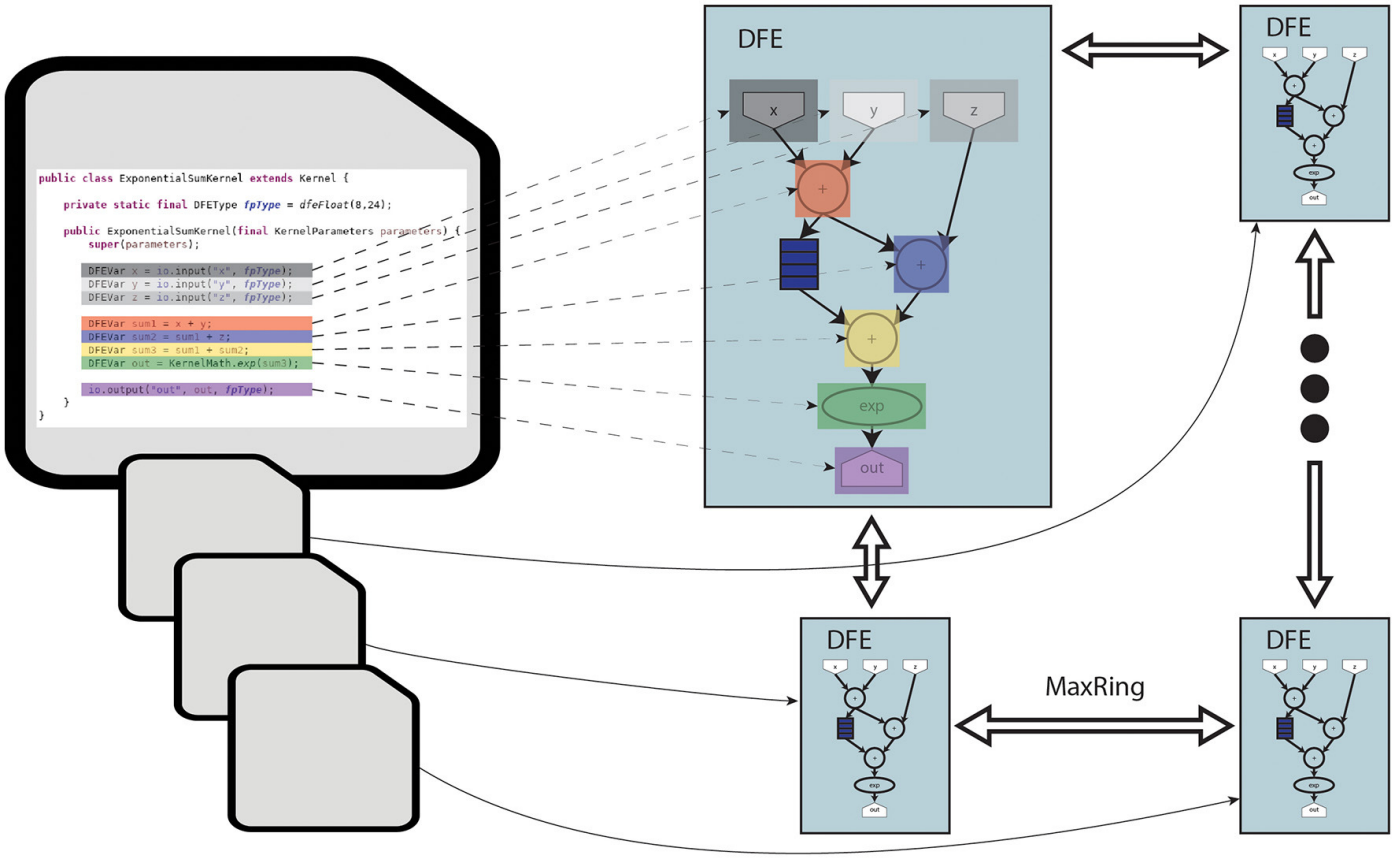
Diagram of MaxJ code being translated to a dataflow graph across multiple DFEs.

The toolflow process begins with the MaxCompiler translating MaxJ kernel code into a dataflow graph. The MaxCompiler uses this graph and the manager description to generate VHDL code. This code is then utilized by FPGA vendor tools for implementation processes like synthesis and place-and-route, ultimately producing a bitfile for use with the C code on the CPU-host. This process is visualized in Figure 3. In this figure, it can be seen that the lines of code are directly translated to functional units on the DFE. Additionally, the figure depicts how multiple DFEs can be connected through MaxRing to construct a larger dataflow graph. Importantly, this graph features a pipelined architecture, thereby significantly enhancing parallelism.

This toolflow significantly simplifies programming complexity compared to traditional low-level hardware-description languages (e.g., VHDL) or even HLS languages (e.g., Vivado C and OpenCL). MaxJ offers more precise control over generated logic, leading to more efficient and optimized design implementation. Thus, the Maxeler toolflow represents an excellent programming environment for efficient development.

### 3.3 *flexHH*

**Figure 4:**
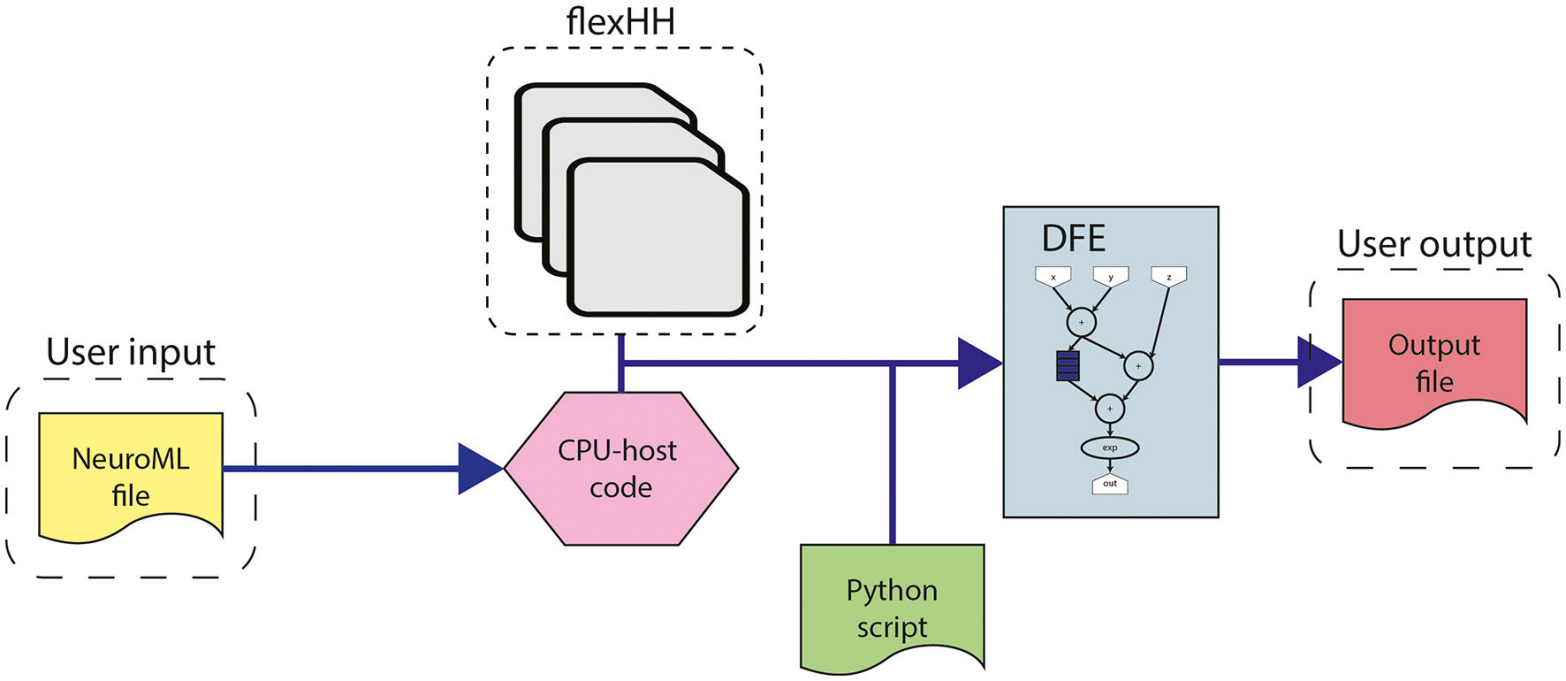
Workflow for the use of *flexHH*.

This work builds upon the original *flexHH* library (Miedema, 2019). *flexHH* is a high-performance, energy-efficient, and flexible hardware library for HH-based simulations. The high performance and energy efficiency primarily originate from the use of the dataflow paradigm on a DFE. However, the library is still easy to use as the workflow presented in Figure 4 shows. The workflow begins with the user input, consisting of parameters of the model, including model parameters such as variables defining the equations, the number of compartments in the network (*N*_*comp*_), and the maximum number of gates per compartment (*N*_*gates*_). These parameters are adaptable to values up to and including the maximum values defined during the hardware synthesis. The model parameters are either sourced from a NeuroML file (automatically parsed into the corresponding values in the CPU-host) or directly inputted from the CPU-host code. Additionally, a scripting language like Python can be used to provide the parameters and execute the precompiled binary. Then these model parameters can be used as input for one of the presynthesized *flexHH* kernels. These kernels which are available as bitstreams, contain the functionality for the simulations of (e)HH models. *flexHH* contains 5 different kernels (*HH, HHg, HHc, HHmc*, and *HHmcg*). Each of the kernels supports a different subset of features [complex ion gates (c), multiple cell compartments (m), and gap junctions (g)]. The naming convention reflects the supported features. For example, *HHmc* supports HH-type models with multiple cell compartments (m) and complex ion gates (c). Therefore, each kernel instance can be somewhat tailored to the user's needs and not waste resources on features not required by the experiment.

**Figure 5:**
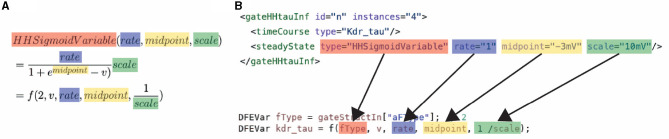
Conversion of a NeuroML function to: **(A)** the mathematical description of the generalized function used in *ExaFlexHH*, and **(B)** a MaxJ function used in the *ExaFlexHH* library.

The high performance and energy efficiency are achieved with the use of the dataflow paradigm via the previously discussed Maxeler tools. Firstly, this is enabled by the use of different kernels, each supporting a different subset of model features, and therefore resources are not wasted on features not required by the simulation. Secondly, this is enabled by the kernels that are flexible and reusable. The flexibility and reuseability are enabled by the generalization of the modeling functions, which are discussed in Section 3.1. Without the equation generalization, a new time-consuming synthesis would be required each time something changes inside the model. Instead, by a generalization of the functions, each constant, parameter, and variable can be set on the CPU host. Consequently, removing the requirement of a new hardware synthesis, thus, resolving one of the main issues for neuroscientists when using an FPGA. An example of how the equations are generalized can be seen in Equation 18. This equation is used to calculate the transition rates within an HH model, Equations (12, 17) in Section 3.1. The input consists of the membrane voltage of compartment *i* (*V*_*i*_), 3 constants (*k*_0_, *k*_1_, *k*_2_), and a variable to select the function branch (*f*_*t*_). An important feature of the equation generalization is the NeuroML compatibility. This compatibility is illustrated in Figure 5, which demonstrates how the NeuroML function HHsigmoidVariable and its parameters can be converted to the f function [which implements Equation (18)], resulting in the exact same functionality. Moreover, as the parameters can be chosen for each cell, compartment and channel separately, *flexHH* supports use cases that most other platforms completely avoid such as heterogeneous eHH networks with gap junctions.


(18) $$f(f_{t},V_{i},k_{0},k_{1},k_{2})=\{\begin{array}{cc} \frac{k_{0}\cdot (k_{1}-V_{i})}{e^{(k_{1}-V_{i})\cdot k_{2}}-1}, & \text{if}\;f_{t}=0\\ k_{0}\cdot e^{(k_{1}-V_{i})\cdot k_{2}}, & \text{if}\;f_{t}=1\\ \frac{k_{0}}{e^{(k_{1}-V_{i})\cdot k_{2}}+1}, & \text{if}\;f_{t}=2 \end{array}$$

The generalization of the equations is done in such a way that it is NeuroML-compatible. Consequently, with the use of a parser, a NeuroML file containing the information of the simulation can be used to define the input parameters of the *flexHH* kernels. Notably, the flexibility and NeuroML compatibility do not compromise performance. The results in the original *flexHH* paper show that *flexHH* outperforms a Xeon Phi 5110P CPU, a Nvidia Titan X GPU, and a hardcoded version on the same DFE for the number of cells supported by *flexHH*. These results indicate the competitive performance of the *flexHH* library on a DFE.

While *flexHH* offers unique benefits, such as high performance and usability, in its current version it has some functional limitations:

- The library exclusively supports serial connections between intracellular compartments. This limitation is justifiable, as numerous models can be supported, given that alternative connection configurations are predominantly necessary for dendritic networks or trees.
- When gap junctions are supported, the amount of calculations will always be $N_{cells}^{2}$ (the amount of calculations required for an all-to-all connected network). Consequently, for networks with less than all-to-all connectivity, this method results in more calculations than optimal.
- The library is limited to the use of a single DFE, constraining its scalability.

For more details about the *flexHH* library, the reader is referred to Miedema (2019).

### 3.4 Implementation

Here, we discuss the implementation of a new hardware library *ExaFlexHH*. Because of the unique benefits of *flexHH* library, this library is extended. We extended its functionality to support the MAX5C DFEs, based on the multi-SLR capable Xilinx Ultrascale+ FPGAs AMD (2023). Additionally, the capability to operate across multiple DFEs has been integrated, facilitated by utilizing the MaxRing technology. The extension is done by taking the code, and thus kernels, from *flexHH* and incorporating the additional functionality so the same equations and workflow can be used. Therefore, the *ExaFlexHH* library will contain the code of the kernels, the CPU-host code, a parser to convert a NeuroML file to the variables used in the CPU-host code, and the Python script to run a simulation. The *ExaFlexHH* library is publicly available in its repository.^1^ *flexHH* includes two categories of kernels (as previously mentioned in Section 3.3): those without gap junctions (*HH, HHc*, and *HHmc*) and those with gap junctions (*HHg* and *HHmcg*). This distinction is made as only the kernels with gap junctions require data communication between the kernels and, thus, additional engineering work. For the sake of simplicity, only the updates to the kernels *HHmc* and *HHmcg* will be discussed in detail, which are individually the most complex designs possible within the *flexHH* and *ExaFlexHH* libraries. Prior to the discussion of the aforementioned kernels, particularly focusing on the data flow and arithmetic, we address the Maxeler Manager, to ensure maintainability and portability, the high-level kernel I/O and kernel instantiation are separated from the hardware-specific details. This is achieved by using a Java interface for the creation of I/O with generic functions and kernel instantiation. The actual managers, which are Java objects, implement the interface and handle hardware-specific details.

#### 3.4.1 Kernels without gap junctions

The *HHmc* kernel calculates the trace of each membrane voltage and gate-activation variable for the requested simulation time. To calculate the membrane voltage (Equation 2) is used, with the exception of *I*_*gap, i*_ as gap junctions are not supported. All of the calculations are local to a cell and can be executed in parallel, allowing for the instantiation of multiple kernels. For the implementation on the MAX5C DFEs, each of which contains three dies, a total of three kernels with one kernel per die were instantiated per device. To use multiple MAX5C DFEs, the kernels on a single device were duplicated. As there is no data communication between the kernels, the performance is bounded by the performance of a single kernel. The original *HHmc flexHH* kernel was found to be limited by the I/O bandwidth of the on-board DRAM.

To analyze this bottleneck, we calculate the required throughput, which can be done accurately due to the deterministic behavior of the kernel. The calculation consists of multiplying the data size of the I/O-stream elements with the required access frequency of these streams, where the access frequency is expressed in number of clock cycles. Furthermore, this product is multiplied by the operating frequency of the DFE. The resulting equation is shown in Equation (19). In this equation, *uf*_*cell*_ is the unroll factor (parameter which unrolls loops in hardware), *N*_*comps, cell*_ the number of compartments per cell, *N*_*gates, comp*_ the number of gates per compartment, and *f* the operational frequency of the kernel. Additionally, the values 96, 32, and 4 (multiple times, for multiple I/O streams) are the resulting values of the data sizes in bytes required at different moments during the execution of the kernel. To reduce the dimensions of the plot, the parameters are fitted to our use case, described in Section 3.1, concerning an IO network.

**Figure 6:**
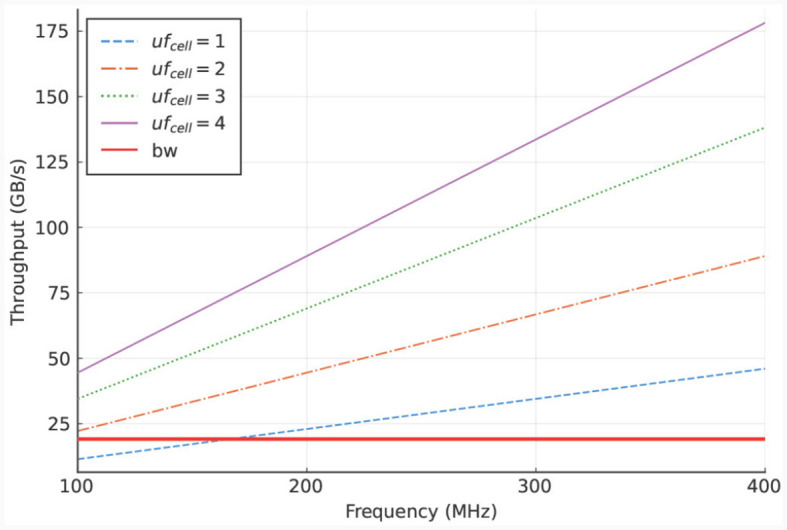
LMem bandwidth (horizontal, red line) and total required throughput for multiple unroll factors (other lines) for a single *HHmc* kernel.

Specifically, *N*_*comps, cell*_ is set to the number of compartments per cell in an IO cell (here: three) and *N*_*gates, comp*_ is set to the minimum number of gates per compartment in the IO model which is also three. The results are shown in Figure 6. These results indicate that the on-board DRAM (called LMem, Large Memory, in the DFE terminology) is a bottleneck for the kernel and that the required throughput exceeds the DRAM's theoretical bandwidth unless the unroll factor *uf*_*cell*_ is set to 1 and the frequency is lower than 168 MHz.


(19) $$\begin{array}{l} \;Throughput_{DRAM,HHmc}=(96+4)\;\cdot \;uf_{cell}\cdot f\\ \;+(32+4+4+4)\cdot \frac{1}{\left\lceil \frac{N_{gates,comp}}{uf_{cell}}\right\rceil }\cdot f\\ \;+4\cdot \frac{1}{\left\lceil \frac{N_{gates,comp}}{uf_{cell}}\right\rceil }\cdot \frac{1}{N_{comps,cell}}\cdot f\\ =(100\;\cdot \;uf_{cell}+(\frac{4}{N_{comps,cell}}+44)\cdot \frac{1}{\left\lceil \frac{N_{gates,comp}}{uf_{cell}}\right\rceil })\cdot f \end{array}$$

#### 3.4.2 Kernels with gap junctions

**Figure 7:**
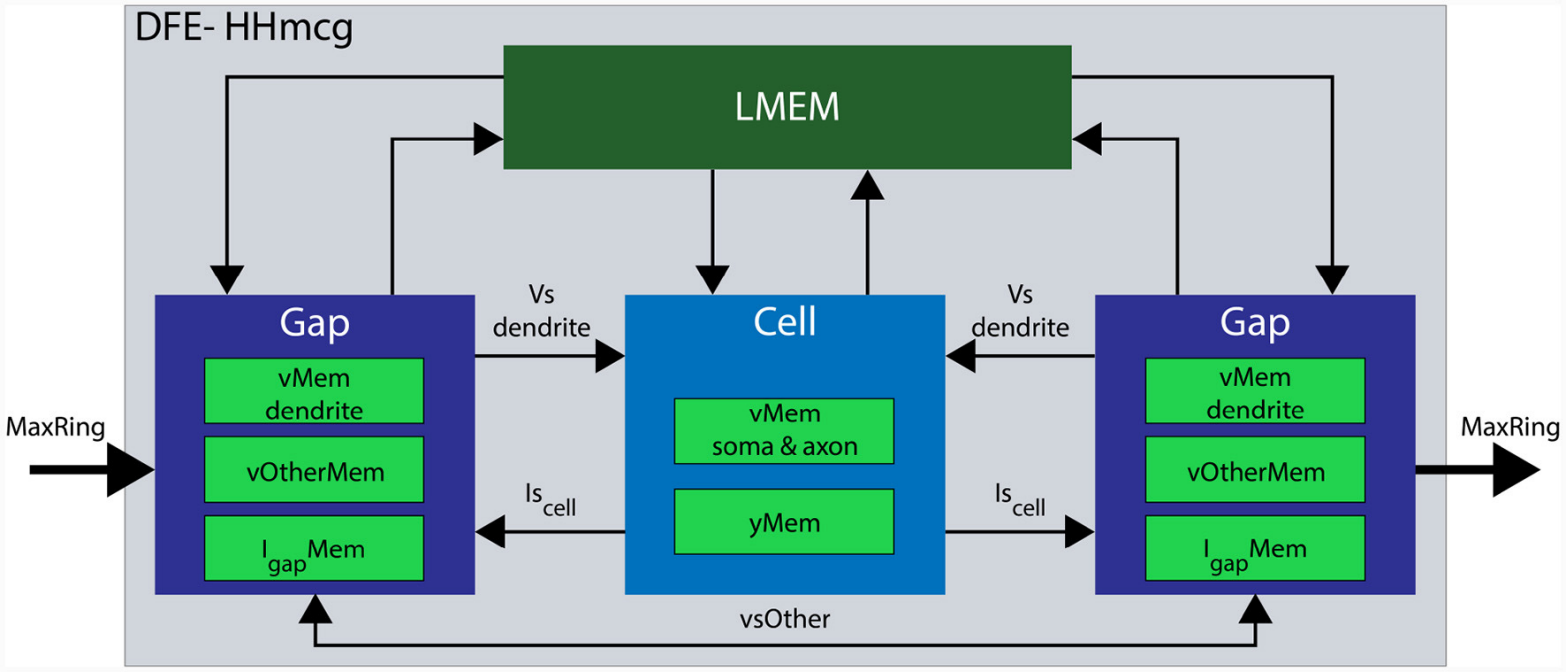
Architectural overview of a single DFE. Data movement is denoted with arrows and memory components are highlighted in various green colors. Data transfer from LMem to the kernels consists of the parameters for both channels and compartments. Conversely, data transfer from the kernel toward the LMem consists of state variables: *vMem* stores membrane voltages, *yMem* stores gate variables, *vOtherMem* stores dendritic voltages of other gap kernels, and *I*_*gap*_*Mem* stores gap-junction currents.

In the updated implementation, the *HHmcg* kernel is divided into two distinct parts: (1) the computations within a cell, and (2) the computations out of the cell, involving gap junctions. As a result, the *HHmcg* kernel is also split into two separate kernels: the *cellCore* kernel and the *gapCore* kernel. The *gapCore* kernel calculates *I*_*gap*_ while the *cellCore* kernel calculates the remainder of the currents of Equation (2) and the dynamics of the gate activation variables. According to Miedema (2019), the computations involving gap junctions are more computationally demanding than those of the inner cell dynamics. Therefore, the *gapCore* kernel plays a crucial role in overall performance. To optimize performance, the computational workload and hardware resources are closely matched. This is achieved by allowing multiple *gapCore* kernels per single *cellCore* kernel, with the number of *gapCore* kernels per cell being variable to support different configurations and future-generation DFEs. An architectural overview of the *HHmcg* kernel is shown in Figure 7, the details will be given when the *gapCore* and *cellCore* kernels are described in more detail in the following section.

##### 3.4.2.1 The *gapCore* kernel

The *gapCore* kernel(s) calculate(s) the gap-junction current *I*_*gap, i*_ for each cell *i* in the network, as described in Equation (6) where *w*_*i, j*_ is the variable connection weight between cell *i* and *j*, *V*_*i*_ the membrane voltage of cell *i* and *c*_0_, *c*_1_, and *c*_2_ are single floating-point constants. To enhance performance, the loop used to calculate *I*_*gap, i*_ is unrolled in hardware using an unroll factor *uf*_*gap*_.

In order to enhance performance, the voltages are stored in the BRAMs. This can be done in either the *gapCore* or *cellCore* kernel. However, similar to the amount of ticks (number of clock cycles which process data in a DFE) required for the computations of the kernel, the amount of memory reads in the *gapCore* kernel scales with the square of the number of cells. On the other hand, the number of reads in the *cellCore* kernel scales with the total number of gates in the entire network. Considering the typical network structure, the number of cells is significantly larger than the number of gates per cell. This assertion is verified by our inspection of the amount of gate variables within the cells of Neuroml-DB (Birgiolas et al., 2023). This inspection showed that the maximum number of gate variables per cell is 24,011, which was an outlier. However, in the perspective of the size of the cerebral cortex, which contains between 10 to 100 million neurons (Braitenberg and Schüz, 2013), it is still relatively small. As a result, the amount of data transfers between the *gapCore* and *cellCore* kernel will be reduced when the dendritic voltages are stored in the gap kernel. In addition to the voltages, the intermediate values of *I*_*gap*_, *i* are also stored in BRAM (*I*_*gap*_*Mem*). To analyze the most efficient way to store the data, the amount of memory blocks required can be calculated. The data-storage requirement is determined by both its width, defined as the number of bits per variable, and its depth, denoting the total number of variables. Given the finite configurations available in hardware, this often results in additional overhead in terms of memory blocks needed. Furthermore, the number of memory blocks required on an FPGA is influenced by the number of read and write ports. This leads to Equation (20) (Voss et al., 2021). This equation describes the number of used memory blocks as the product of the required data width divided by the width in hardware (*w*), the required data depth divided by the depth in hardware (*d*), and the number of read and write ports divided by the number of ports available in hardware (*p*). From this equation it follows that it is advantageous to store data in vectors as the reduction in depth and number of write ports outweigh the increase in data width.


(20) $$\begin{array}{l} n_{mem}=\left\lceil \frac{w_{req}}{w_{hardware}}\right\rceil \times \left\lceil \frac{d_{req}}{d_{hardware}}\right\rceil \times \left\lceil \frac{p_{req}}{p_{hardware}}\right\rceil \end{array}$$

Unrolling or vectorizing the calculations within the *gapCore* kernel is a method to leverage the hardware capabilities of a DFE to facilitate more concurrent operations, thereby optimizing the performance of a single DFE. Additionally, multiple *gapCore* kernels can be run in parallel on MAX5 cards to further enhance performance. The voltages of each cell are stored in the BRAMs, and the intermediate values of the gap-junction current are also stored there. By using multiple identical kernels, the summation of the gap-junction current can be split into multiple, equal-sized calculations, allowing for the same code to calculate the gap-junction currents to be used across all kernels while still selecting the correct data. This is possible because the gap junctions are all-to-all connected as is shown in Equation (6). Let *N*_*gap*−*kernels*_ be the total number of *gapCore* kernels. Then, each kernel stores $\frac{N_{cells}}{N_{gap-kernels}}$ voltages. This amount will further be referred to as *N*_*cells, gap*_. Furthermore, we introduce *Vs*_*gap, k*_, which is the array containing the voltages stored in *gapCore* kernel *k*. With this variable, we can rewrite Equation (6) to clarify the requirement of data transfer when multiple kernels are used. This is shown in Equation (21) where there are 4 *gapCore* kernels. From this equation, it becomes clear that the summation to calculate the gap-junction current can be split into multiple, of equal size and equal functionality, summations. Consequently, the same code can be used to instantiate multiple kernels with the same functionality. However, the right data is required to be selected from memory. Namely, it has to be decided if either *Vs*_*gap*, 0_, *Vs*_*gap*, 1_, *Vs*_*gap*, 2_, or *Vs*_*gap*, 3_ has to be used. Therefore, we introduce the variable *vsOther*. This variable will hold *Vs*_*gap*, 0_, *Vs*_*gap*, 1_, *Vs*_*gap*, 2_, or *Vs*_*gap*, 3_ based on which phase of the program is being executed.


(21) $$\begin{array}{l} I_{gap,i}=\sum_{j=0\;}^{N_{Cells}/(N_{gap-kernels=4)}}(w_{i,j}(c_{0}\exp(c_{1}\cdot (V_{i}-Vs_{gap,0}[j]{)}^{2})+c_{2})(V_{i}-Vs_{gap,0}[j]))\\ \;+\sum_{j=0}^{N_{Cells}/(N_{gap-kernels=4)}}(w_{i,j}(c_{0}\exp(c_{1}\cdot (V_{i}-Vs_{gap,1}[j]{)}^{2})+c_{2})(V_{i}-Vs_{gap,1}[j]))\\ \;+\sum_{j=0}^{N_{Cells}/(N_{gap-kernels=4)}}(w_{i,j}(c_{0}\exp(c_{1}\cdot (V_{i}-Vs_{gap,2}[j]{)}^{2})+c_{2})(V_{i}-Vs_{gap,2}[j]))\\ \;+\sum_{j=0}^{N_{Cells}/(N_{gap-kernels=4)}}(w_{i,j}(c_{0}\exp(c_{1}\cdot (V_{i}-Vs_{gap,3}[j]{)}^{2})+c_{2})(V_{i}-Vs_{gap,3}[j])) \end{array}$$

The data transfer in the proposed system is designed to only occur between neighboring kernels, as dictated by the ring topology. Therefore, this architecture implements a one-dimensional systolic array. To ensure that all data is correctly sent to each kernel, the system is divided into three different phases:

1. **Start:** During the initial phase of the program, partial calculations of the gap-junction currents are performed using the locally stored voltages in the kernel. These voltages are then sent to the neighboring kernels that require them for further calculations. This occurs concurrently with the calculations themselves.
2. **Middle:** During this phase, the gap-junction currents have already been updated with the influence of the local kernel's voltages. The received voltages, stored in the *vsOther* vector, are then used to further update the gap-junction currents. Concurrently, the voltages are sent to the next *gapCore* kernel. Because the voltages require to pass through each DFE, this phase may consist of multiple stages, where the gap-junction currents are updated and sent to the next neighboring kernel in each stage.
3. **End:** In the final phase, the final voltages are received and the final values of the gap-junction currents are calculated. The voltages are then fully updated.

**Figure 8:**
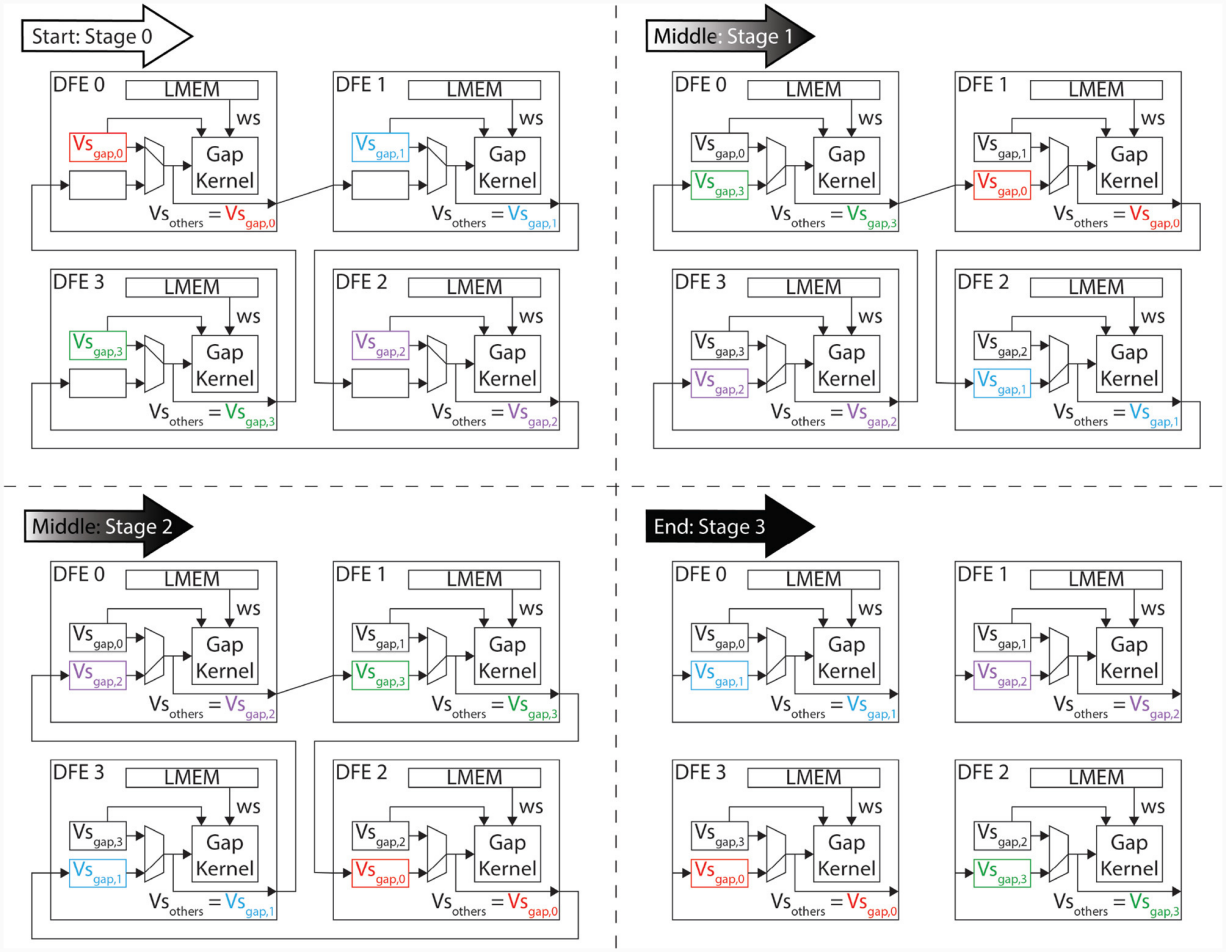
Program flow in *ExaFlexHH* when instantiating 4 gap-junction kernels on multiple DFEs.

The execution flow is visualized in Figure 8, which shows an example with four gap kernels on 4 different DFEs. However, the schedule is flexible and works regardless of the number of *gapCore* kernels. Moreover, this schedule is flexible and successful for both inner and inter DFE data transfers. The implementation does not differentiate between data received from another DFE via MaxRing or from another die on the same DFE. Namely, as is shown in Algorithm 1, the kernel will receive its data from a general input and send it to a general output function, which both are agnostic of the source or target of the data. Based on the configuration, the data will be transferred to the appropriate kernel.

**Algorithm 1:**
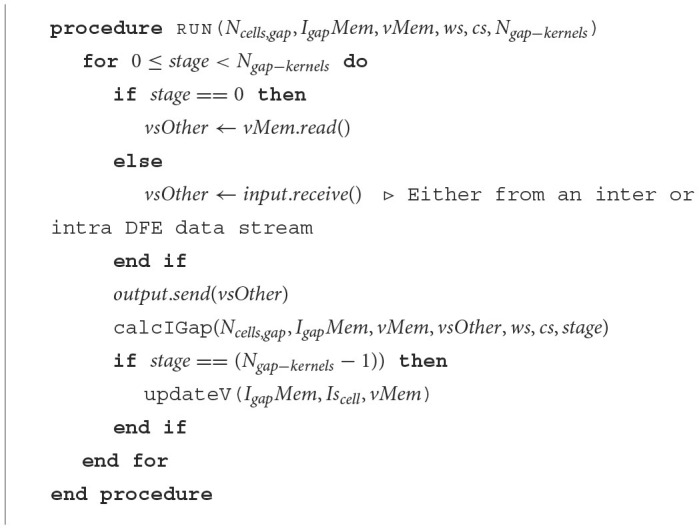
Pseudocode of the program flow of a single gapCore kernel.

In addition to data transfer, the *gapCore* kernel implements the functions calcIGap and updateV. The function calcIGap calculates the gap-junction currents according to Equation (21). Therefore, it accepts as input parameters (i) the number of cells per gap kernel *N*_*cells, gap*_, (ii) the memory *I*_*gap*_*Mem* for storing the intermediate values of the currents, (iii) the memory *vMem* containing the voltages stored in the respective *gapCore* kernel, (iv) *vsOther*, the dendritic voltages obtained from other DFEs, stored in *vOtherMem*, (v) the weights *ws* of the connectivity matrix, (vi) the constants *cs* [*c*_0_, *c*_1_, and *c*_2_ from equation Equation (6)], and (vi) the variable *state* tracking in which state the algorithm is. The updateV function updates the voltages of the respective kernel in the final stage using the forward-Euler numerical method. Therefore, it accepts as input parameters (i) memory *I*_*gap*_*Mem* containing currents of the gap junctions, (ii) the currents *Is*_*cell*_ from the inner cell dynamics, and (iii) the dendritic voltages from *vMem*, to retrieve the current values of these state variables and update them accordingly. The currents from the inner cell dynamics *Is*_*cell*_ must be obtained from the *cellCore* kernel.

Because the data transfers occur exclusively between neighboring kernels, any number of kernels can be chained together in a ring topology, thereby, achieving a scalable hardware implementation. Furthermore, by transmitting all the data at the start of each stage, the time available for data transfer is maximized, thereby minimizing the required throughput. The amount of ticks required per stage can be calculated via Equation (22). In this equation *loopLength*_*gap*_ is the pipeline depth in the *gapCore* kernel, *N*_*cells, gap*_ is the number of cells per *gapCore* kernel, and *uf*_*gap*_ the unroll factor of the *gapCore* kernel. Subsequently, the throughput requirements can be determined via Equation (23). In this equation *size*_*data*_ is the size of the data which is required to transfer over the MaxRing during a stage, and *N*_*ticks, stage*_ the ticks per stage as described in the previous equation.


(22) $$N_{ticks,stage}=\left\{ \begin{array}{l} loopLength_{gap}\cdot N_{cells,gap},\text{ if }loopLength_{gap}>N_{iterations}\\ \frac{N_{cells,gap}^{2}}{uf_{gap}},\text{ otherwise} \end{array}\right.$$

where


(23) $$\begin{array}{lll} \;N_{iterations} & = & \frac{N_{cells,gap}}{uf_{gap}}\\ Throughput_{MaxRing,gap} & = & \frac{size_{data}\cdot f}{N_{ticks,stage}} \end{array}$$

**Figure 9:**
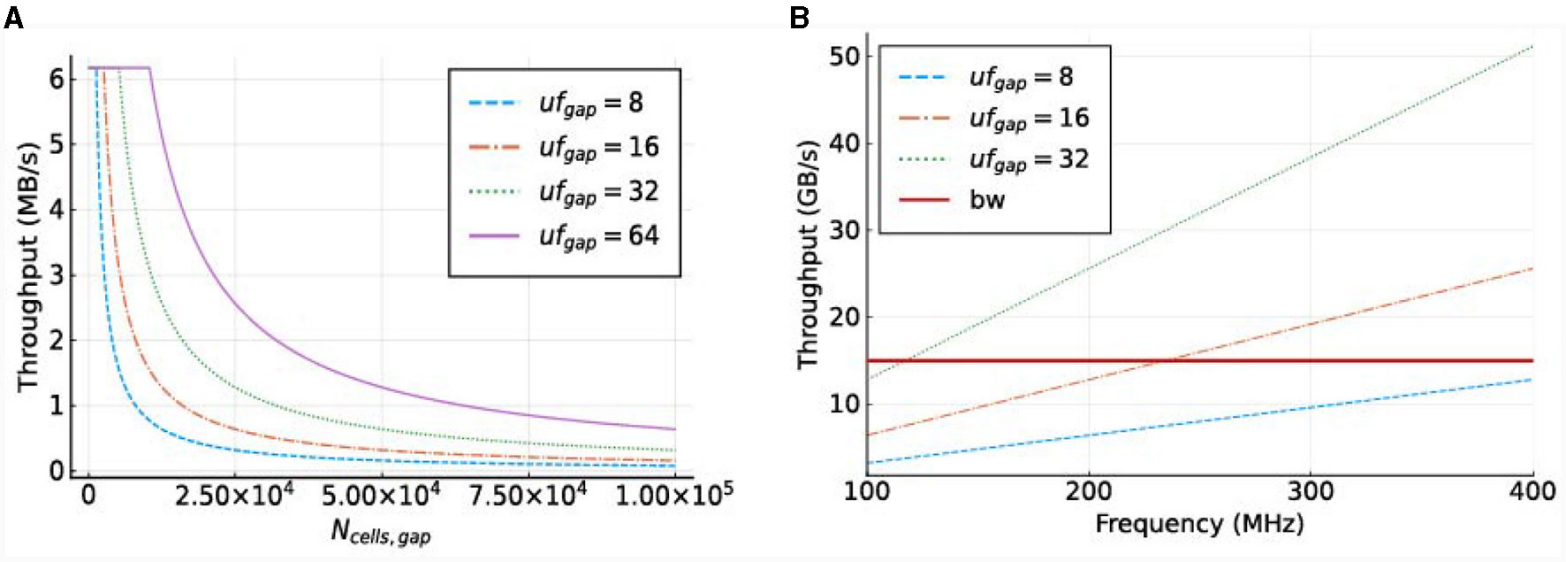
Required throughput for the *gapCore* kernel. **(A)** Required MaxRing throughput for different *gapCore*-kernel unroll factors, at a frequency of *f* = 250*MHz*. **(B)** On-board DRAM bandwidth (horizontal, red line) and total required throughput for multiple unroll factors (other lines) for a single *gapCore* kernel.

To illustrate the required throughput, a frequency of 250*MHz* is used, as this is the maximum frequency achievable by MAX5 DFEs according to the Maxeler documentation. The required throughput is visualized in Figure 9A. This demonstrates that the MaxRing bandwidth, at 5 Gbyte/s, will not be a performance bottleneck, as the required throughput is significantly lower.

The potential performance bottlenecks in our system are the bandwidth of the on-board DRAM and the amount of compute resources available. As discussed previously, each SLR has its own DRAM DIMM, and a single *gapCore* kernel is implemented on a single Super Logic Region (SLR). Therefore, the available DRAM bandwidth is that of a single DIMM, which is 15 GB/s. The gap kernel has three streams connected to the on-board DRAM: (1) an input stream for the initial values of the voltages (*vsIn*); (2) an input stream for the weights of the gap-junction connections (*ws*), which are stored in a connectivity matrix, and (3) an output stream to store the values of the voltages at each simulation time step (*vsOut*). The values of *vsIn* are only streamed toward the kernel at the start of each simulation, and the voltages are then stored in the BRAMs. The stream for *vsOut* is only active in the last iteration of each time step. Both of these streams are negligible in comparison to the duration of the I/O stream of weights *ws*, which are used and streamed constantly throughout execution. Therefore, only the throughput requirements for the weights are used for the analysis of the on-board DRAM data transfers. This gives a required throughput of 4·*uf*_*gap*_·*f* bytes. The required throughput for the *gapCore* kernel is shown in Figure 9B. The figure shows that for an unroll factor of 32 and a frequency higher than 118*MHz*, the required throughput exceeds the theoretically available DRAM bandwidth. Additionally, this also holds for an unroll factor of 16 and frequencies higher than 236*MHz*. However, in practice, the effective bandwidth is expected to be lower. Consequently, it is expected that the performance of the gap kernel will be bounded by the bandwidth of the on-board DRAM.

In the previously discussed implementation, the weights for the gap junctions are stored in the on-board DRAM of the DFEs. Therefore, during simulation these weights need to be transferred between the DRAM and the DFE. To improve performance beyond what the DRAM bandwidth permits, an alternative approach is proposed: rather than loading the weights from the on-board DRAM, which demands more memory bandwidth with a higher *uf*_*gap*_, we can have the weights of the gap junctions generated directly on the DFE itself during simulation. These weights (ranging from 0.0 to 1.0) can be sampled from a hardware kernel on the DFE which implements one or more stochastic processes. Such an approach is permissible in this context of realistic neural simulations where large-scale network dynamics are often initialized based on some stochastic distribution, as exemplified in Negrello et al. (2019). In effect, avoiding DRAM use will significantly reduce the throughput requirement between the on-board DRAM and the DFE.

To achieve this, the generation of the connectivity weights is implemented as separate and independent kernels, promoting modularity. However, due to the direct relationship between the number of calculations and limited hardware resources, a limited number of generation schemes, that require these calculations and corresponding hardware, are supported. In this work, we support the generation of weights based on the uniform and Gaussian distributions, as previously shown in literature; for instance, the book by Braitenberg and Schüz (1998) and the works of Pfeuty et al. (2003) and Knight and Nowotny (2021).

For the implementation of random-number generation, the Squares algorithm (Widynski, 2020) is used to generate pseudo-random numbers with a uniform distribution. This algorithm is efficient for implementation on the DFE due to its counter-based nature. To generate random numbers that follow a Gaussian distribution, the uniform numbers generated by the Squares algorithm are put through the *probit* function.

##### 3.4.2.2 The *cellCore* kernel

The functionality of the *cellCore* kernel is similar to that of the *HHmc* kernel, however, there are several modifications required due to the interaction with the gap kernels. These modifications include:

- The addition of an input stream to receive voltages from the *gapCore* kernels, which are used to calculate the inner-cell dynamics of compartments with gap-junctions.
- The addition of output streams to send calculated currents and elastances (inverse of the capacitance) to the *gapCore* kernels. These values are needed for the calculations in the *gapCore* kernels.
- Additional control logic is implemented to determine if a compartment connected to a gap junction is being processed. This control is required to manage the input and output streams, as well as to prevent writing to the BRAMs of voltages of compartments connected to gap-junctions.
- The data between the *gapCore* and *cellCore* kernel must be vectorized or non-vectorized to match the different data processing methods used in each kernel. For vectorization, shift registers are used, and for non-vectorization, counters and multiplexers are used to select the correct data from a vector.

## 4 Results

In this section, the performance and scalability of *ExaFlexHH* using the *HHmc* and *HHmcg* kernels will be evaluated. To this end, kernel execution times, FLOPS (floating-point operations per second), and energy results are presented. The section begins with a description of the experimental setup in Section 4.1. Subsequently, the results of the *HHmc* are discussed in Section 4.2, followed by a discussion of the results of the *HHmcg* kernel in Section 4.3.

### 4.1 Experimental setup

The De Gruijl IO model, with and without gap junctions, is utilized as a case study to assess the kernel performance. In the variant with gap junctions, the network is characterized by an all-to-all connectivity, which is employed to maximize the load on the interconnect, as this network type necessitates the most data transfer. A presentation of this model was given in Section 3.1.

For the kernel execution, we utilize the Maxeler tools and multiple Max5C DFEs the specifications of which are shown in Table 2. These DFEs are connected through the MaxRing in a ring topology and are also connected to a CPU host, an Intel Xeon Bronze 3104 CPU with a frequency of 1.70GHz, via two InfiniBand links, providing a bandwidth of 14 GB/s. Furthermore, to instantiate multiple *HHmc* kernels, MPI is used. This limits the amount of parallel MPI jobs as it depends on the number of available CPU cores. Therefore, for the particular host CPU, a maximum of 6 parallel MPI processes can be launched, and although 8 DFEs are available only 6 could be used when running multiple *HHmc* kernels. Because the *HHmc* kernels do not communicate with each other, MPI is only used to spawn multiple processes and thus kernels. For the *HHmcg* kernels we can use the max_run_array function from the Maxeler tools. This function facilitates the creation of a DFE array, including one with 8 DFEs. Consequently, here we are not confined to the MPI process limit of 6.

**Table 2:** Specifications of the multi-DFE platform, where *ExaFlexHH* is deployed.

**Specification**	**Value**
DFE model	MAX5C
Chip architecture	Xilinx Ultrascale+
LUTs (k)	1,182
FFs (k)	2,364
DSPs	6,840
On-chip memory capacity (MB)	43.2
On-board DRAM DIMMs	3
On-board DRAM capacity per DIMM (GB)	16
On-board DRAM bandwidth per DIMM (GByte/s)	15
SLRs	3
MaxRing bandwidth (GByte/s)	5
Host CPU	Intel^®^ Xeon^®^ Bronze 3104
MaxelerOS version	2021.1
MPI version	Open MPI 3.1.3

Kernel execution times are measured using the gettimeofday() function on the CPU host, and the number of floating-point operations per IO cell is determined through kernel profiling. The FLOPS are calculated by considering the number of operations used in the generalized functions, similar to those in *flexHH*, as discussed in Section 3.3. Therefore, some functions may use more operations than necessary in a hardcoded scenario. To simplify the analysis, all operations are considered in the calculation of FLOPS. This decision is based on two reasons: (1) the FLOPS are consistent across all kernels used to simulate the IO, allowing for a clear comparison of performance scaling when using different numbers of DFEs and kernels, and (2) the hardware for the generalized equations is allocated even if it is not always utilized, making it a reliable measure of performance. The number of operations is multiplied by weight factors as presented in Thant et al. (2005) and by the number of ticks in a single step, and finally multiplied by the number of kernels to obtain the total number of FLOPS. To measure power consumption, the maxtop command from the Maxeler toolflow is used. The maxtop command presents the power consumption based on sensors present on the DFEs themselves.

The results for both the *HHmc* and *HHmcg* kernels include the execution time per step for different numbers of cells, the number of FLOPS achieved for single and multiple DFEs, and the energy efficiency in the form of FLOPS/W. The value of *N*_*gates, max*_ is set to 6, representing the maximum number of gates per compartment. The unroll factor for the *cellCore* kernel, *uf*_*cell*_, is set to 1, which is the maximum value before exceeding the bandwidth of the on-board DRAM. Meanwhile, the unroll factor for the *gapCore* kernel, *uf*_*gap*_, is set to 16, being the maximum value that correctly synthesizes. The subsequent feasible value is 32 (owing to the necessity for powers of two due to data alignment on the DFE) but this would demand an excessive amount of hardware resources, consequently leading to failed synthesis. The maximum number of compartments *N*_*comps, max*_ that can be successfully synthesized with a frequency of 170*MHz* is 57,344. This gives the resource usage as presented in Table 3. This shows the availability of hardware resources. However, as previously discussed, larger unroll factors did not lead to performance improvements or did not synthesize correctly. Moreover, the implementation where the connectivity weights are generated on the DFE itself could not be synthesized correctly with more unrolling than the original implementation. Therefore, the results of the implementation where the connectivity weights are generated on the DFE itself are excluded.

**Table 3:** Resource utilization of the *HHmc* and *HHmcg* kernels on the Max5C DFE.

	**Max5C**	**HHmc**	**HHmcg**
LUTs	1,182,240	241,879 (20.5%)	230,463 (19.5%)
FFs	2,364,480	438,890 (18.6%)	472,169 (20.0%)
BRAMs	4,320	4,320 (30.0%)	2,290 (69.2%)
URAMs	960	209 (21.5%)	288 (30.0%)
DSPs	6,840	453 (6.6%)	1,181 (17.3%)

### 4.2 HHmc

**Figure 10:**
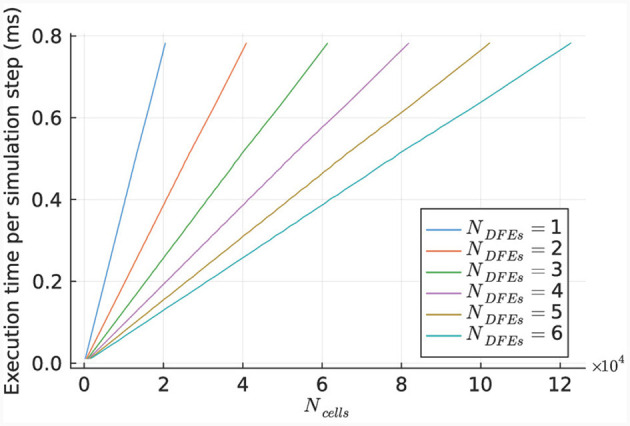
Execution time per step of the *HHmc* kernel, as measured for different sizes of IO networks.

The performance of the kernel for various numbers of cells is illustrated in Figure 10, using 1 to 6 DFEs. The primary limitation in kernel performance is the bandwidth of the on-board DRAM. Hence a larger unroll factor for the *cellCore* kernel does not lead to better performance. The results indicate a linear relationship between the execution time and the number of simulated cells, as expected due to the parallel nature of the cell computations.

**Figure 11:**
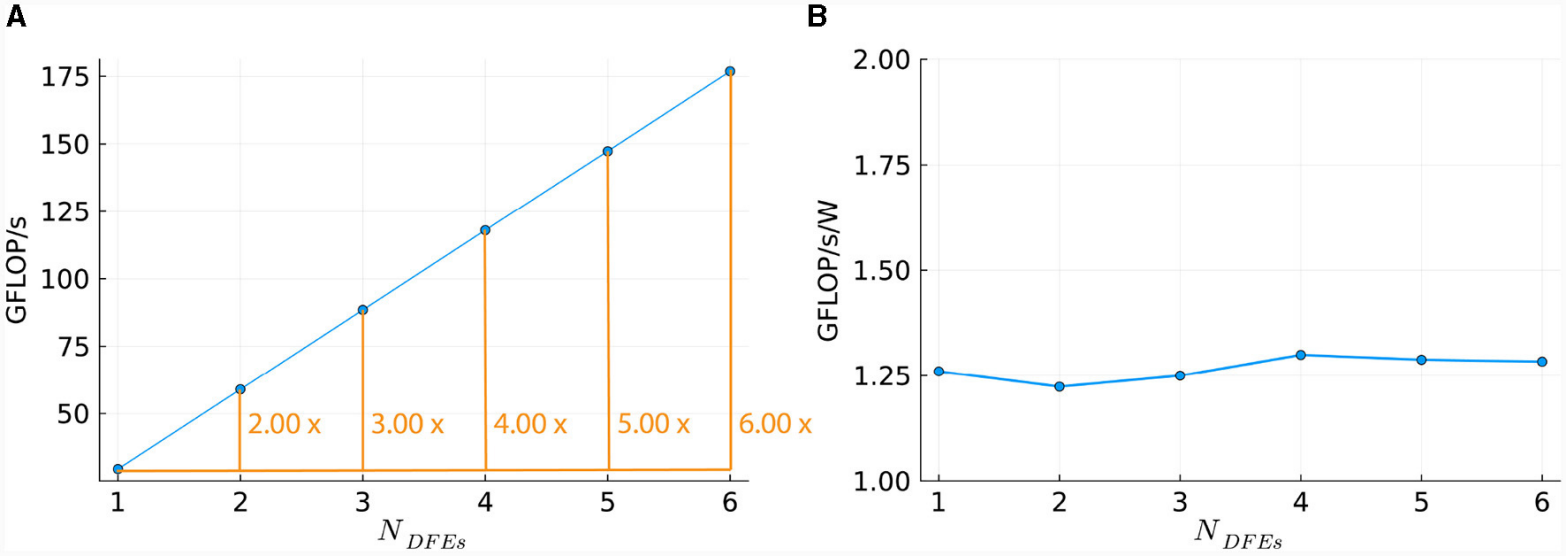
Scaling of *HHmc* kernel on multiple DFEs, as measured in terms of: **(A)** performance; **(B)** energy efficiency.

This linear relationship is also reflected in the number of DFEs used and the GFLOPS achieved, as shown in Figure 11A. Power consumption was measured (results are presented in Supplementary material) and used to calculate energy efficiency in GFLOPS per Watt, as shown in Figure 11B. These results show a small variation which is also observed in the power usage of the DFEs when in idle state. Consequently, the results follow our expectations and therefore, can provide a reference point for the performance of the *HHmcg* kernels that require communication; to be discussed next.

### 4.3 HHmcg

**Figure 12:**
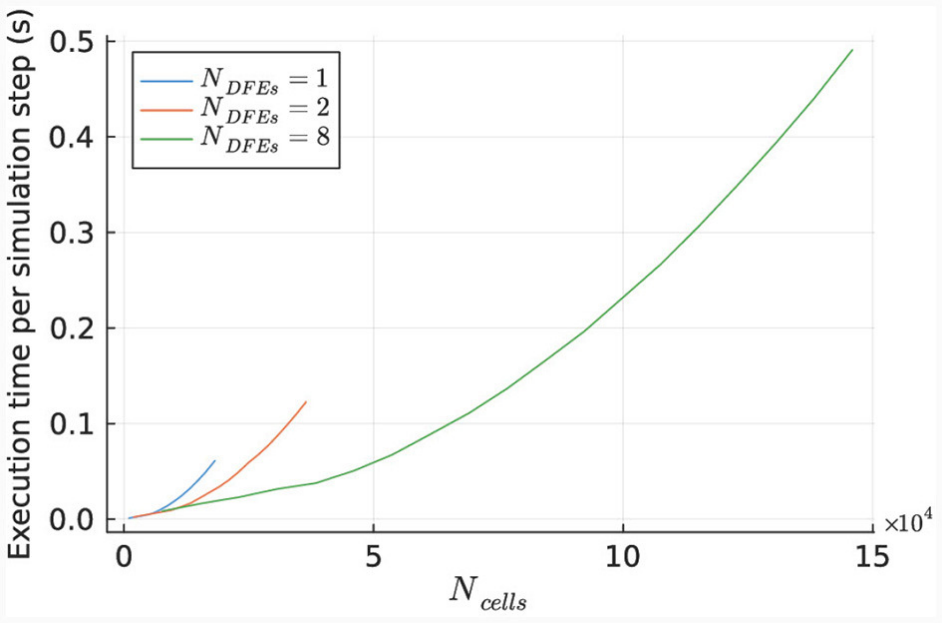
Execution time per step of the *HHmcg* kernel, as measured for different sizes of IO networks.

The performance of the *HHmcg* kernel is evaluated for the use of 1, 2, and 8 DFEs. The execution time per simulation step of the kernel is measured for various network sizes and is shown in Figure 12. The results indicate a linear scaling for a low number of cells, which is because that the dataflow pipeline of the performance-critical kernel of the gap junctions on the DFE is not fully saturated with data. This leads to empty stages within the pipeline and unused hardware resources.

More specifically, the pipeline is completely filled and utilized when there are enough voltages to divide over all the *uf*_*gap*_ pipelines within the *gapCore* kernel. This is captured by the inequality *N*_*cells, gap*−*kernel*_·*uf*_*gap*_ ≥ *d*_*gap*_, where *N*_*cells, gap*−*kernel*_ is the number of cells per *gapCore* kernel, *uf*_*gap*_ the unroll factor of the *gapCore* kernel, and *d*_*gap*_ the depth of the pipeline of the *gapCore* kernel. To provide a better overall indication of when all the pipelines are fully utilized, the inequality is rewritten as *N*_*cells, total*_·*uf*_*gap*_ ≥ *d*_*gap*_·*N*_*DFEs*_·*N*_*gap*−*kernels, DFE*_, where *N*_*cells, total*_ is the total number of cells, *N*_*DFEs*_ is the number of DFEs, and *N*_*gapkernels, DFE*_ is the number of *gapCore* kernels per DFE.

The linear relationship between the execution time and the number of cells is explained by the fact that for each iteration, each cell being processed is still required to pass through the complete pipeline. Furthermore, there are $\frac{N_{cells,gap-kernel}}{uf_{gap}}$ iterations per step, as is described in section 3.4.2. Consequently, the number of ticks (number of clock cycles which process data in a DFE) for a simulation of *N*_*steps*_ in the partially filled pipeline is equal to the first branch of Equation (24). Here *N*_*gapkernels, total*_ denotes the number of total *gapCore* kernels. On the other hand, in case enough data is present to completely fill the pipeline, each membrane voltage of the cells in the *gapCore* kernels needs to be processed in each iteration for a simulation of *N*_*steps*_. Therefore, in this case, the number of ticks of the kernel scales quadratically with the number of cells, as can be seen in the second branch of Equation (24).


(24) $$N_{ticks,HHmcg}=\left\{ \begin{array}{l} N_{steps}\cdot d_{gap}\cdot N_{cells,gap-kernel}\cdot N_{gapkernels,total},\text{ if }N_{cells,gap-kernel}\cdot uf_{gap}\geq d_{gap}\\ N_{steps}\cdot N_{cells,gap-kernel}^{2}\cdot N_{gapkernels,total}÷uf_{gap},\text{ otherwise} \end{array}\right.$$

**Figure 13:**
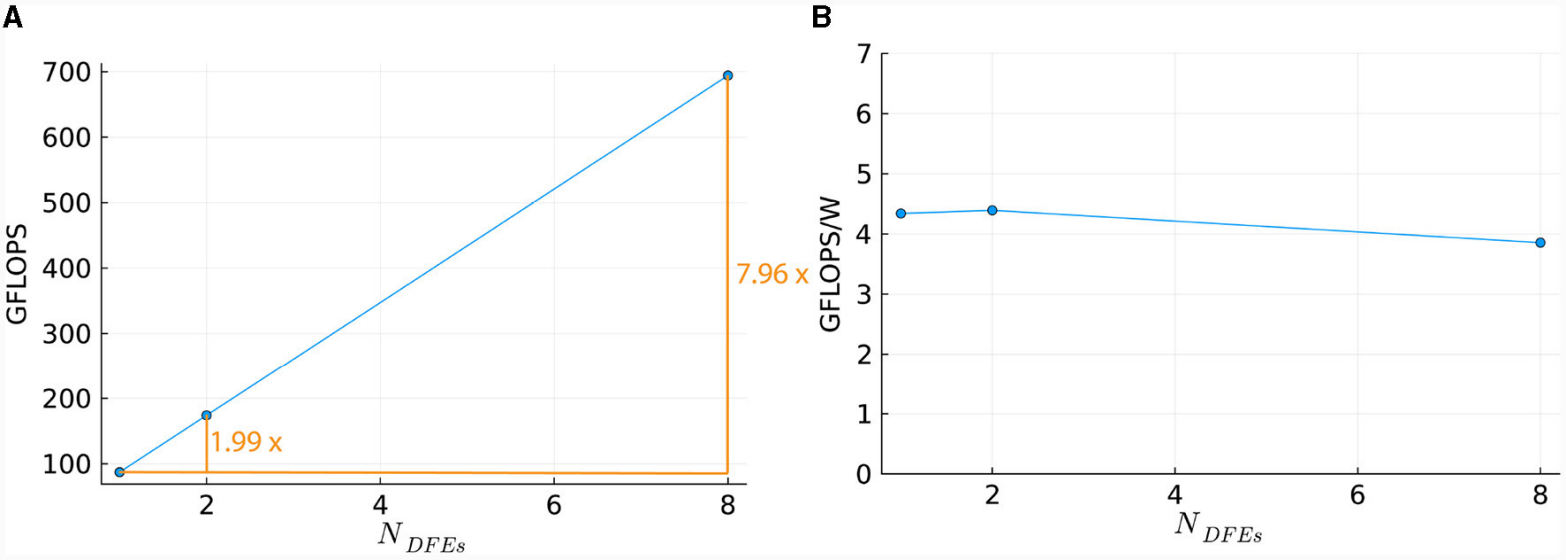
Scaling of the *HHmcg* kernel on multiple DFEs, as measured in terms of: **(A)** performance; **(B)** energy efficiency.

To compare the performance of using different numbers of DFEs, the timing results of the maximum number of cells are used to calculate the FLOPS. These results can be seen in Figure 13A: increasing the number of DFEs to 2 or 8 leads to an increase of 1.99 and 7.97 in computational power, respectively, thus showing excellent performance scalability. We can conclude that the performance and performance scalability are not bounded by the MaxRing interconnect due to its ultralow-overhead design. This is not typical of multi-node setups. For illustrative purposes, we compared the performance scalability of our implementation against two other works simulating an IO network: a multi-GPU setup supporting GPUDirect, as detailed in Vlag et al. (2019), and a multi-node many-core CPU architecture as described in Chatzikonstantis et al. (2019). The results presented in Table 4 demonstrate superior performance scalability for *ExaFlexHH*. Notably, *ExaFlexHH* maintains a consistent performance trend, particularly when the DFEs are fully utilized with data. In contrast, the work in Vlag et al. (2019) reported a variable speedup, dropping to as low as 8 × under similar simulations. Furthermore, Chatzikonstantis et al. (2019) in fact reported a decrease in performance during scaling out in their experiments for the case of uniform connectivity distributions and a connectivity density of 1,000 synapses per neuron, which is much lower than that supported by *ExaFlexHH*.

**Table 4:** Comparison of performance scaling between *ExaFlexHH*, the work presented in Vlag et al. (2019) and Chatzikonstantis et al. (2019).

**Implementation**	**Accelerator**	**Maximum number of accelerators**	**Connectivity**	**Maximum speedup**	**Scaling efficiency (%)**
*ExaFlexHH*	Maxeler MAX5C DFE	8	nCells^2^ (all-to-al)	7.97	99.63
Vlag et al. (2019)	Nvidia Tesla K40M GPU	32	1000 · nCells (uniform distributed)	24.00	75.00
Chatzikonstantis et al. (2019)	Intel Xeon Phi Knights Landing	8	1000 · nCells (uniform distributed)	1.48	18.50

*ExaFlexHH* is connected in a ring forming a *one-dimensional systolic array*. This architecture inherently supports the expansion with an arbitrary number of DFEs. Such scalability combined with the excellent scaling performance of *ExaFlexHH* indicates that there is no theoretical limitation of the performance potential of *ExaFlexHH*. This implies the feasibility of *ExaFlexHH* to achieve exascale performance. However, practically the number of DFEs is limited and to reach exascale performance the performance of a single node should be increased. The performance of a single DFE is determined by both the hardware resources and the bandwidth of the on-board DRAM. Specifically for *ExaFlexHH*, the performance is limited by the *GapCore* kernel. The performance of this kernel will increase with sufficient hardware resources to increase the unroll factor *uf*_*gap*_ to the next feasible value of 32 (owing to the necessity for powers of two due to data alignment on the DFE) and more on-board DRAM to facilitate the corresponding throughput. A prediction of the achievable performance of *ExaFlexHH* will be discussed in Section 5.2.

Power measurements were done (results are presented in Supplementary material) to calculate the energy efficiency, represented by the FLOPS per Watt. The energy efficiency is shown in Figure 13B. The energy efficiency is near-constant, with a variance within 0.3 GFLOPS/W. This is as expected as variance within power usage is also present when the DFEs are in an idle state. Consequently, we can conclude that the observed excellent performance scalability does not compromise system energy efficiency.

## 5 Discussion

In this section, we will attempt a comparison between *ExaFlexHH* and other FPGA-based implementations from related works in terms of (normalized) performance (Section 5.1). Furthermore, we will construct an analytical performance model of *ExaFlexHH* and will use it to attempt performance projections based on future technologies (Section 5.2). As previously stated in Section 3.4, the performance of both the *gapCore* and *cellCore* kernels was expected to be limited by the bandwidth of the on-board DRAM in the target system, and the results have confirmed this bottleneck. However, with advancements in technology, such as HBM, memory bandwidth is expected to increase significantly in the future. Despite lagging behind other HPC platforms, such as GPUs, in terms of DRAM bandwidth, there are no inherent technological limitations for FPGAs. The difference can be attributed to market forces and the target market of FPGA manufacturers. As the FPGA industry starts to focus on the HPC domain, the performance of FPGAs is expected to progress in the future (Shahzad et al., 2021).

### 5.1 Comparison against other FPGA implementations

To evaluate the performance of *ExaFlexHH* against related works, a comparison is made with other FPGA-based neural simulation implementations introduced and discussed in Section 2. To ensure a fair and comprehensive comparison, the following aspects are considered: neural-model complexity, FPGA-device characteristics, and (normalized) performance. An overview of the comparison is presented in Table 5.

**Table 5:** Overview of competitive FPGA-based brain simulation implementations.

**Hardware implementation**	**BlueHive**	**SNAVA**	**NeuroFlow**	**ExaFlexHH (this work)**
Model	Izhikevich	Izhikevich	Izhikevich	IO (extended HH)
Synapse type	Chemical	Chemical	Chemical	Electrical (gap junctions)
Connectivity *(# synapses/neuron)*	1,000	10	10,000	all-to-all
Network size *(# cells)*	256,000	2,000	589,824	145,920
Precision	16-bit fixed-point	16-bit fixed-point	Single floating-point	Single floating-point
FPGA chip	Stratix IV 230	Kintex-7 XC7K325T	Stratix V 5SGSD8	Virtex UltraScale+ VU9P
Number of FPGAs	4	2	6	8
Device capacity *(# LUTs or # ALMs)*	91,200 ALMs	595,096 LUTs	262,400 ALMs	1,182,000 LUTs
Performance *(G[FL]OPS)*	5.89	25.84	16.42	694.11
Resource efficiency *([FL]OPS/LUT)*	8,070	43,426	5,214	73,404

In demonstrating the results of eHH models with gap junctions, the library *ExaFlexHH* is a unique solution for the simulation of these models on multiple DFEs. The models simulated by BlueHive, SNAVA, and NeuroFlow are Izhikevich models with chemical synapses, which are simpler than the IO model and not biologically plausible as discussed in Section 2. This simplicity leads to less computations required for simulating the dynamics, which affects the maximum network size that can be simulated. The larger network sizes for BlueHive and NeuroFlow can be explained by the simpler models used.

Another factor that affects the amount of hardware required is the precision of the implementations. BlueHive and SNAVA use 16-bit fixed point variables, which require less hardware but sacrifice accuracy compared to single floating-point variables. To make a fair comparison, the performance density is used, represented by the FLOPS per processing element. In this case, the processing element is defined as a 6-input Look-Up Table (LUT) or an Adaptive Logic Module (ALM). Because some FPGAs use ALMs instead of LUTs we adopt the assumption that 2 ALMs ≈ 4 6-input LUTs from Smaragdos et al. (2014).

The FLOPS of the related works are calculated using the execution times from their respective papers and the number of FLOPS per neuron (Izhikevich, 2004). For chemical synapses, 1 FLOP per active synapse is assumed, as they can be represented by the accumulation of weights, as shown in the BlueHive implementation (Moore et al., 2012). For *ExaFlexHH*, the results from Section 4 are repeated.

The comparison shows that *ExaFlexHH* achieves an order of magnitude higher performance compared to the related work, and a *resource efficiency* between 1.69 and 14.08 times higher. The high performance allows for large-scale simulations of biophysically plausible models, even when the neural network is fully connected with gap junctions, which require continuous interaction and limit parallelism. The high resource efficiency is indicative of the employed dataflow paradigm and resulting, systolic-array-like, operation of the platform and is telling of the *ExaFlexHH* benefits.

### 5.2 Performance model and future potential

The results in Section 4 suggest no inherent limitations to scaling our architecture to exascale performance. Beyond scaling, advancements in hardware can also increase performance. To demonstrate the future performance potential of *ExaFlexHH* with evolving hardware, an analytical performance model can be created. The model ought to be highly accurate given the deterministic nature of the *ExaFlexHH* library.

The execution time of the kernel is calculated using Equation (25), where *T*_*comp*_ represents the computational time, determined by the product of the number of ticks (*N*_*ticks*_) and the frequency (*f*) of the kernel, as shown in Equation (26), and *T*_*DRAM*_ represents the time to transfer data between the kernel and the on-board DRAM, which can be computed with Equation (27) by dividing the total amount of data to be transferred (*D*_*DRAM*_) by the bandwidth (*BW*_*DRAM*_). Here the amount of data required to be transferred is equal to the size required to be transferred of the data per tick (*size*_*data, tick*_) multiplied by the *N*_*ticks*_ and *f*. *T*_*MaxRing*_ represents the time to transfer data between DFEs over the MaxRing, but as discussed in section 3.4.2, the MaxRing is not expected to be a bottleneck for performance, as confirmed by the excellent scalability results in Section 4.3. Thus, for the sake of brevity, *T*_*MaxRing*_ will be omitted from the performance model as its impact is minimal. Furthermore, for the purpose of this discussion it is assumed that all designs will have access to adequate hardware resources on future FPGAs and will meet timing constraints, thereby ensuring correct synthesis.


(25) $$\begin{array}{ll} T_{exec} & =max\left(T_{comp},T_{DRAM},T_{MaxRing}\right) \end{array}$$

(26) $$\begin{array}{l} \text{where}\\ T_{comp}\;=\frac{N_{ticks}}{f} \end{array}$$

(27) $$\begin{array}{ll} T_{DRAM} & =\frac{D_{DRAM}}{bw_{DRAM}}=\frac{size_{data,tick}\cdot N_{ticks}\cdot f}{bw_{DRAM}} \end{array}$$

In the following, we will present the performance model of both the *cellCore* and *gapCore* kernels. As depicted in Equations (26, 27), the computational time and the time required for data transfer between the kernel and DRAM scale linearly with the frequency. Without loss of generality, we will set the frequency to 170*MHz* for this analysis.

#### 5.2.1 The *cellCore* kernel

**Figure 14:**
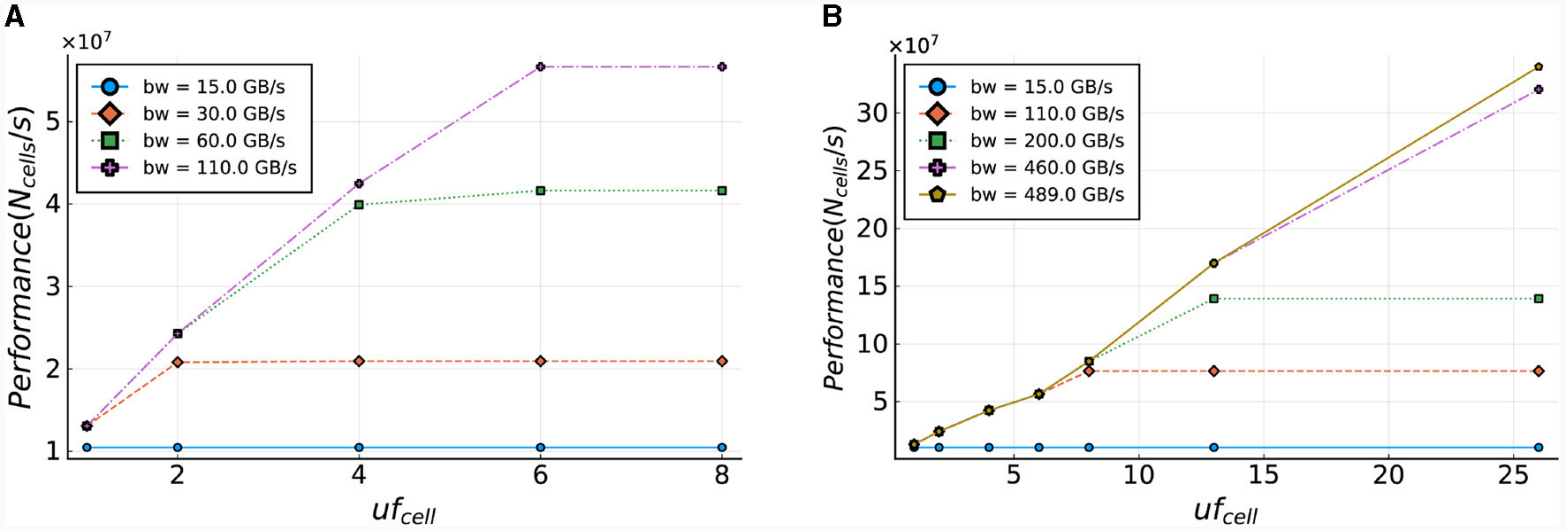
Estimated performance of the *cellCore* and *HHmc* kernel. Performance is calculated as the number of cells (single step) that can be processed within a second. **(A)** Performance projections based on the current *ExaFlexHH* version. **(B)** Performance projections for a modified version of *ExaFlexHH* with support for processing multiple compartments and cells in parallel.

The computation of the number of ticks required for the *cellCore* kernel (*N*_*ticks, cell*_) is performed using Equation (28). In this equation, *N*_*steps*_ is the number of simulation steps, *N*_*comps, total*_ represents the total number of compartments in the whole model, and *N*_*gates*_[*i*] is the number of gates of compartment *i*. Additionally, *uf*_*cell*_ is the unroll factor of the *cellCore* kernel, and *size*_*buffer*_ is the size of the buffer required for maintaining a variable inner loop. For a more elaborate discussion of this buffer, the reader is referred to Miedema (2019). This equation computes the execution time needed for a single time-step of one cell, for various bandwidths, and is used to determine the number of single time-step calculations of IO cells that can be processed per second. The results are presented in Figure 14A and demonstrate that the *cellCore* kernel is limited by the bandwidth of the on-board DRAM, as previously discussed in Sections 3.4.1, 4.2. The figure also showcases the improvement in performance as the DRAM bandwidth increases (e.g., when moving to HBM-type memory), reaching its maximum throughput of 110 GB/s. It is important to note that increasing the unroll factor from six to eight has no effect on performance, as the maximum number of gates in a compartment of an IO cell is six, resulting in any additional hardware remaining idle.


(28) $$\begin{array}{l} N_{ticks,cell}=N_{steps}\sum_{i=1}^{N_{comps,total}}\left\lceil \frac{N_{gates}[i]}{uf_{cell}}\right\rceil +size_{buffer} \end{array}$$

To further enhance performance, *ExaFlexHH* must be expanded to enable parallel processing of compartments and cells, in addition to gates. Figure 14B presents the performance potential for this expanded framework, assuming the use of better on-board memory bandwidth (up to realistic limits of current HPC accelerators) and increased hardware resources. With support for parallel processing of multiple compartments (*uf* = 8 and *uf* = 13, where a single IO cell contains 13 gates in *ExaFlexHH*), the figure demonstrates an increase in potential performance as the unroll factor increases. Furthermore, when *uf* = 26, or when two IO cells are processed in a single tick, performance continues to improve with the parallel processing of more data. The model shows that performance will continue to increase until a maximum bandwidth of 490 GB/s is reached, assuming sufficient hardware resources are available. These results highlight the potential benefits of using high-bandwidth memory technologies in *ExaFlexHH*, such as HBM with a bandwidth of 460 GB/s, providing future-proofing potential for the kernel.

#### 5.2.2 The *gapCore* kernel

**Figure 15:**
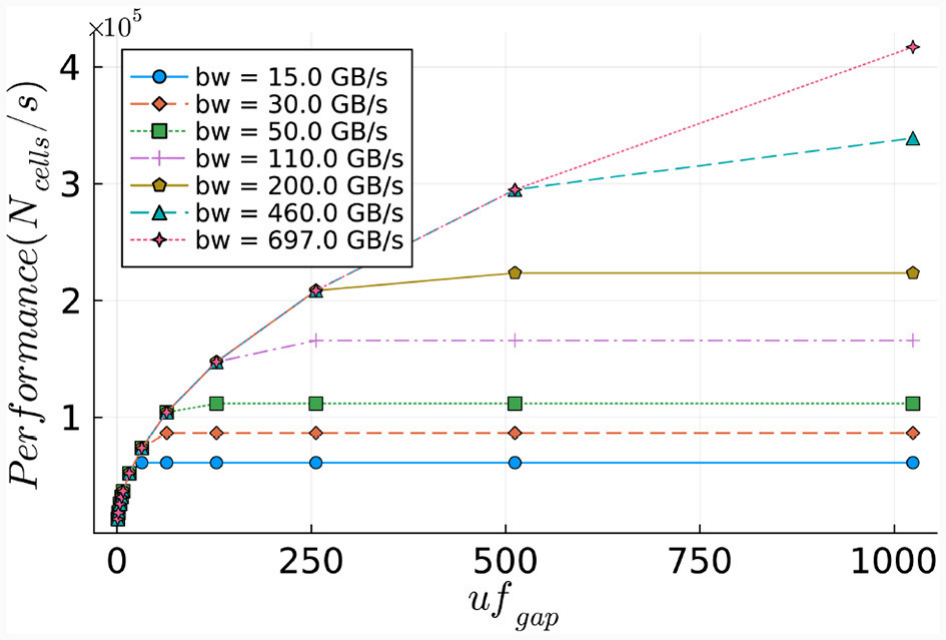
Estimated performance potential of the *gapCore* kernel. Performance is calculated as the number of cells (per single simulation step) that can be processed within a second.

The number of ticks required for the execution of the *gapCore* kernel is calculated via Equation (29). Similar to the *cellCore* kernel, this equation is used to calculate the execution time required for one time-step for various bandwidths. The results, presented in Figure 15, demonstrate the relationship between the memory bandwidth and the maximum network size that can be calculated within a second. As anticipated, the *gapCore* kernel benefits significantly from higher memory bandwidths, given that sufficient hardware resources are available for the calculations. The results indicate that performance increases up to a maximum throughput of 697 GB/s for an unroll factor of 1,024. Additionally, the results display a quadratic increase in both the number of ticks and memory size, due to the $N_{cells}^{2}$ elements in the connectivity matrix.


(29) $$\begin{array}{l} N_{ticks,gap}=N_{steps}\cdot \frac{N_{cells}^{2}}{uf_{gap}} \end{array}$$

## 6 Conclusion

In this work, we have addressed the formidable challenge of simulating large-scale, complex, biologically plausible eHH networks, focusing on advanced connectivity modeling. To achieve this, we conducted a comprehensive assessment of various multi-hardware accelerator platforms, evaluating their performance and scalability, flexibility, usability, and model support. High performance is a critical factor in rendering the simulation of biologically plausible networks viable, while scalability is imperative for accommodating larger and more intricate neural models. Additionally, an ideal platform should be flexible enough to adapt to the evolving demands of model development, and user-friendly, to eliminate the need for extensive hardware and programming expertise. Unfortunately, none of the established platforms appear to fully meet these requirements for the target model complexity of our work.

Our novel solution, the *ExaFlexHH* hardware library, has been engineered to address these shortcomings, building upon the foundation of the flexible and user-friendly *flexHH* library, used for single-node simulations. It allows for seamless modification of neural parameters, encompassing gate and membrane properties, the number of compartments, and the quantity of cells, all without the time-consuming process of hardware (re)synthesis. Furthermore, *ExaFlexHH* accommodates heterogeneous neuron models and is designed to be NeuroML-compliant, with a future goal of developing a NeuroML parser.

One of the distinctive features of *ExaFlexHH* is its ability to facilitate communication between gap-junction kernels and cell kernels on different accelerator devices, enabling multi-device support. Its design has been meticulously optimized to leverage hardware parallelism, and it offers expandability, allowing for the addition of a greater number of DFEs. The hardware configuration and algorithmic design are modular, ensuring ease of maintenance and portability.

Our performance evaluations demonstrate that *ExaFlexHH* exhibits linear performance scalability, measured in GFLOPS, particularly in scenarios without gap junctions, as indicated by the performance results of the *HHmc* kernel. Even in the presence of gap junctions, it showcases nearly linear scalability, exemplified by the results of the *HHmcg* kernel. Specifically, employing two DFEs instead of one results in a performance increase of approximately 1.99, and eight DFEs yield an impressive factor of 7.96 in enhanced performance. Notably, our results reveal consistent performance efficiency in GFLOPS per watt, suggesting significant potential for harnessing emerging DFE/FPGA hardware with minimal porting efforts. In conclusion, *ExaFlexHH* represents a high-performance, scalable, and future-proof multi-FPGA simulation solution specifically tailored for eHH models, addressing the demanding requirements of modern neuroscientific research. Its highly scalable nature facilitates exascale-ready computing speeds, further enhancing its utility in pushing the boundaries of future brain-simulation platforms.

Future work in *ExaFlexHH* should aim to broaden its neural-model support by extending the current features and addressing the current limitations. This entails, among other things, accommodating additional model types, integrating chemical synapse support, extending the support for different compartmental cell structures, and the optimization of lower gap-junction connectivities. Furthermore, we propose to collaborate with neuroscientists to provide them a powerful tool while simultaneously receiving practical feedback.

## Data availability statement

The original contributions presented in the study are included in the article/Supplementary material, further inquiries can be directed to the corresponding authors.

## Author contributions

RM: Conceptualization, Investigation, Methodology, Software, Writing – original draft, Writing – review & editing. CS: Conceptualization, Funding acquisition, Resources, Supervision, Writing – original draft, Writing – review & editing.

## Footnotes

^1^ https://gitlab.com/c7859/neurocomputing-lab/Inferior_OliveEMC/ExaFlexHH
