# Supplementary material for: ExaFlexHH: an exascale-ready, flexible multi-FPGA library for biologically plausible brain simulations
Source: Front Neuroinform. 2024 Apr 12;18:1330875. doi: 10.3389/fninf.2024.1330875 (PMC11045893; doi:10.3389/fninf.2024.1330875)
Supplement: Supplementary file 1 [file Data_Sheet_1.pdf]

Supplementary Material

1 EQUATIONS OF THE INFERIOR-OLIVE MODEL

Below the equations of Inferior-Olive model as defined in De Gruijl et al. (2012) can be found. This model is used to benchmark *ExaFlexHH*.

$$\frac{dV_{dend}}{dt} = \frac{-I_{gap} + I_{app} - I_{sd} + I_{cah,d} + I_{kca,d} + I_{h,d} + I_{leak,d}}{C_{m,d}} \tag{S1}$$

where:

$$\begin{aligned}
i &= \text{number current cell} \\
j &= \text{number other cell} \\
V_{i,j} &= V_{dend,i} - V_{dend,j} \\
I_{gap_i} &= \sum_{j=0}^{N-1} (w_{i,j} \cdot (0.8 \cdot e^{-0.01 \cdot V_{i,j}^2} + 0.2) \cdot V_{i,j}) \\
I_{app} &= \text{input current, can vary per simulation step} \\
I_{sd} &= \frac{g_{int}}{1 - p_1} \cdot (V_{dend} - V_{soma}) \\
I_{cah,d} &= g_{cah,d} \cdot r_d^2 \cdot (V_{cah,d} - V_{dend}) \\
I_{kca,d} &= g_{kca,d} \cdot s_d \cdot (V_{kca,d} - V_{dend}) \\
I_{h,d} &= g_{h,d} \cdot q_d \cdot (V_{h,d} - V_{dend}) \\
I_{leak,d} &= g_{leak,d} \cdot (V_{leak,d} - V_{dend})
\end{aligned}$$

$$\frac{dr_d}{dt} = 0.2(\cdot \frac{1.7}{1 + e^{-\frac{V_{dend} - 5}{13.9}}} \cdot (1 - r_d) - \frac{0.1 \cdot \frac{V_{dend} + 8.5}{-5}}{1 - e^{\frac{V_{dend} + 8.5}{5}}} \cdot r_d) \tag{S2}$$

$$\frac{ds_d}{dt} = min(0.00002 \cdot Ca2Plus_d, 0.01) \cdot (1 - s_d) - 0.015 \cdot s_d \tag{S3}$$

$$\frac{dq_d}{dt} = \frac{\frac{1}{1 + e^{\frac{V_{dend} + 80}{4}}} - q_d}{\frac{1}{e^{-0.086 \cdot V_{dend} - 14.6}} + e^{0.070 \cdot V_{dend} - 1.87}} \tag{S4}$$

$$\frac{dCa2Plus_d}{dt} = -3 \cdot I_{cah,d} - 0.075 \cdot Ca2Plus_d \tag{S5}$$

$$\frac{dV_{soma}}{dt} = -\frac{I_{ds} - I_{as} + I_{cal,s} + I_{na,s} + I_{kdr,s} + I_{k,s} + I_{leak,s}}{C_{m,s}} \tag{S6}$$

where:

$$\begin{aligned}
I_{ds} &= \frac{g_{int}}{p_1} \cdot (V_{soma} - V_{dend}) \\
I_{as} &= \frac{g_{int}}{1 - p_2} \cdot (V_{soma} - V_{axon}) \\
I_{cal,s} &= g_{cal,s} \cdot k_s^3 \cdot l_s \cdot (V_{cal,s} - V_{soma}) \\
m_s &= \frac{1}{1 + e^{-\frac{V_{soma} + 30}{5.5}}} \\
I_{na,s} &= g_{na,s} \cdot m_s^3 \cdot h_s \cdot (V_{na,s} - V_{soma}) \\
I_{kdr,s} &= g_{kdr,s} \cdot n_s^4 \cdot (V_{kdr,s} - V_{soma}) \\
I_{k,s} &= g_{k,s} \cdot x_s^4 \cdot (V_{k,s} - V_{soma}) \\
I_{leak,s} &= g_{leak,s} \cdot (V_{leak,s} - V_{soma})
\end{aligned}$$

$$\frac{dk_s}{dt} = \frac{1}{1 + e^{-\frac{(V_{soma} + 61)}{4.2}}} - k_s \tag{S7}$$

$$\frac{dl_s}{dt} = \frac{\dfrac{1}{1 + e^{-\frac{V_{soma} + 85.5}{-8.5}}} - l_s}{\dfrac{20 \cdot e^{\frac{V_{soma} + 160}{30}}}{1 + e^{\frac{V_{soma} + 84}{7.3}}} + 35} \tag{S8}$$

$$\frac{dh_s}{dt} = \frac{\dfrac{1}{1 + e^{\frac{V_{soma} + 70}{5.8}}} - h_s}{3 \cdot e^{\frac{V_{soma} + 40}{-33}}} \tag{S9}$$

$$\frac{dn_s}{dt} = \frac{\dfrac{1}{1 + e^{-\frac{V_{soma} + 3}{10}}} - n_s}{5 + 47 \cdot e^{\frac{-(-50 - V_{soma})}{900}}} \tag{S10}$$

$$\frac{dx_s}{dt} = \frac{1.3 \cdot \frac{V_{soma} + 25}{10}}{1 - e^{-\frac{V_{soma} + 25}{10}}} \cdot (1 - x_s) - 1.69 \cdot e^{\frac{V_{soma} + 35}{-80}} \cdot x_s \tag{S11}$$

$$\frac{dV_{axon}}{dt} = -\frac{I_{sa} + I_{na,a} + I_{k,a} + I_{leak,a}}{C_{m,a}} \tag{S12}$$

where:

$$I_{sa} = \frac{g_{int}}{p_2} \cdot (V_{axon} - V_{soma})$$

$$m_a = \frac{1}{1 + e^{-\frac{V_{axon} + 30}{5.5}}}$$

$$I_{na,a} = g_{na,a} \cdot m_a^3 \cdot h_a \cdot (V_{na,a} - V_{axon})$$

$$I_{k,a} = g_{k,a} \cdot x_a^4 \cdot (V_{k,a} - V_{axon})$$

$$I_{leak,a} = g_{leak,a} \cdot (V_{leak,a} - V_{axon})$$

$$\frac{dh_a}{dt} = \frac{\frac{1}{1 + e^{-\frac{V_{axon} + 60}{-5.8}}} - h_a}{1.5 \cdot e^{\frac{V_{axon} + 40}{-33}}} \tag{S13}$$

$$\frac{dx_a}{dt} = \frac{1.3 \cdot \frac{V_{axon} + 25}{10}}{1 - e^{-\frac{V_{axon} + 25}{10}}} \cdot (1 - x_a) - 1.69 \cdot e^{\frac{V_{axon} + 35}{-80}} \cdot x_a \tag{S14}$$

REFERENCES

De Gruijl, J. R., Bazzigaluppi, P., de Jeu, M. T. G., and De Zeeuw, C. I. (2012). Climbing fiber burst size and olivary sub-threshold oscillations in a network setting. *PLOS Computational Biology* 8, 1–10. doi:10.1371/journal.pcbi.1002814
