# Supplementary figures and images for: ExaFlexHH: an exascale-ready, flexible multi-FPGA library for biologically plausible brain simulations
Source: Front Neuroinform. 2024 Apr 12;18:1330875. doi: 10.3389/fninf.2024.1330875 (PMC11045893; doi:10.3389/fninf.2024.1330875)

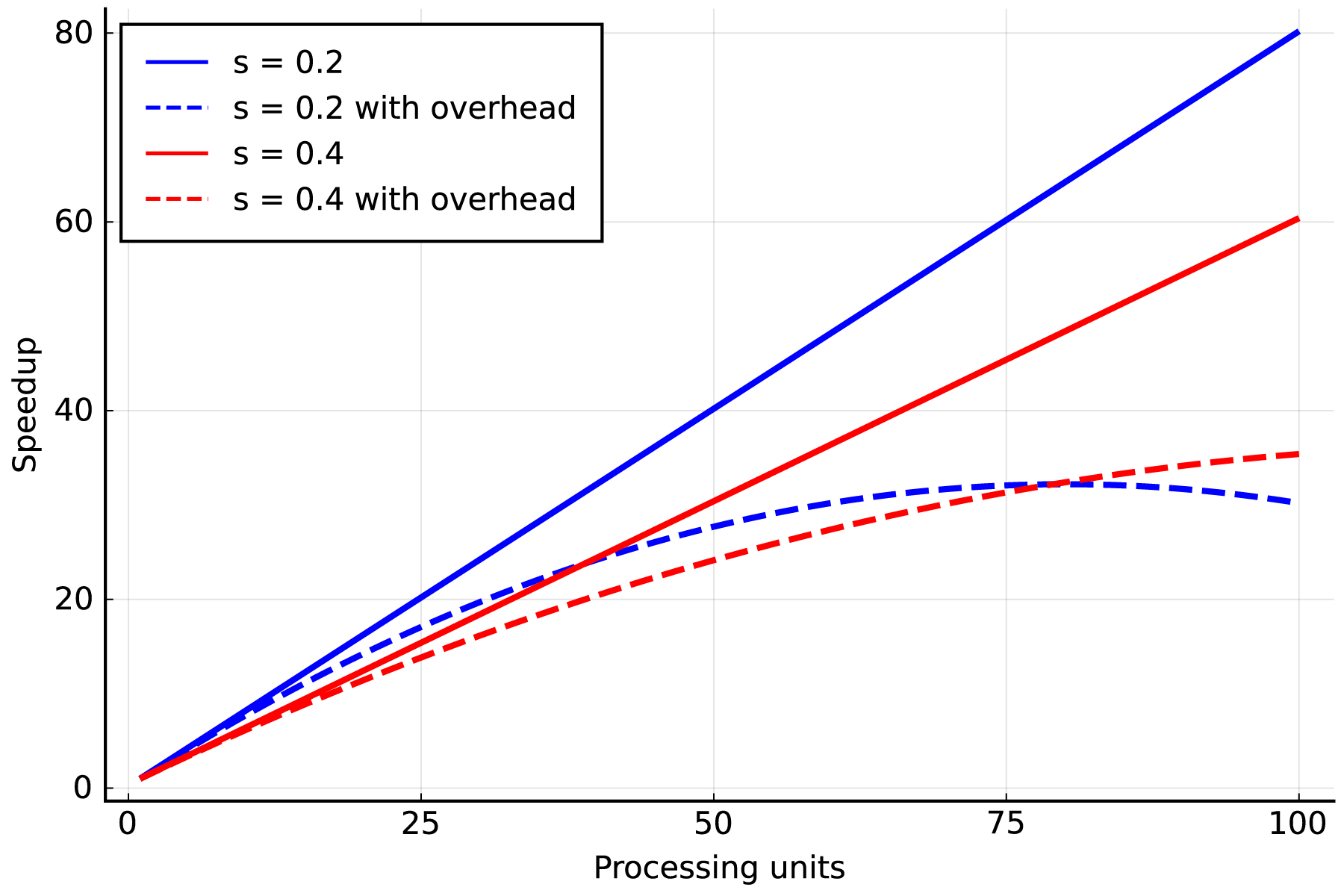

Supplement: Supplementary file 2 [file Image_1.JPEG]

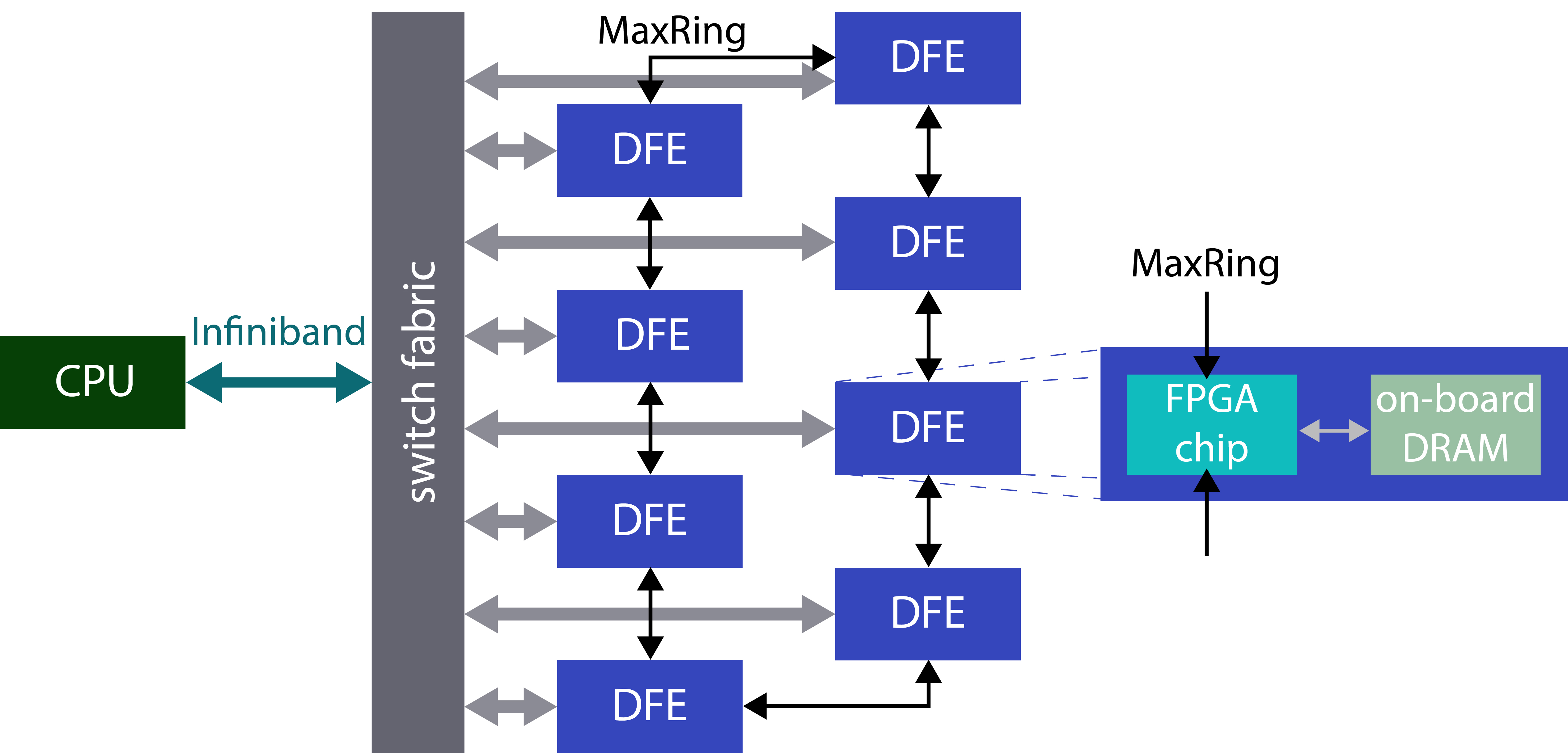

Supplement: Supplementary file 3 [file Image_2.JPEG]
